# Multilevel selection favors fragmentation modes that maintain cooperative interactions in multispecies communities

**DOI:** 10.1371/journal.pcbi.1008896

**Published:** 2021-09-13

**Authors:** Gil J. B. Henriques, Simon van Vliet, Michael Doebeli

**Affiliations:** 1 Department of Zoology, University of British Columbia, Vancouver, Canada; 2 Biozentrum, University of Basel, Basel, Switzerland; 3 Department of Mathematics, University of British Columbia, Vancouver, Canada; Max-Planck-Institute for Evolutionary Biology, GERMANY

## Abstract

Reproduction is one of the requirements for evolution and a defining feature of life. Yet, across the tree of life, organisms reproduce in many different ways. Groups of cells (e.g., multicellular organisms, colonial microbes, or multispecies biofilms) divide by releasing propagules that can be single-celled or multicellular. What conditions determine the number and size of reproductive propagules? In multicellular organisms, existing theory suggests that single-cell propagules prevent the accumulation of deleterious mutations (e.g., cheaters). However, groups of cells, such as biofilms, sometimes contain multiple metabolically interdependent species. This creates a reproductive dilemma: small daughter groups, which prevent the accumulation of cheaters, are also unlikely to contain the species diversity that is required for ecological success. Here, we developed an individual-based, multilevel selection model to investigate how such multi-species groups can resolve this dilemma. By tracking the dynamics of groups of cells that reproduce by fragmenting into smaller groups, we identified fragmentation modes that can maintain cooperative interactions. We systematically varied the fragmentation mode and calculated the maximum mutation rate that communities can withstand before being driven to extinction by the accumulation of cheaters. We find that for groups consisting of a single species, the optimal fragmentation mode consists of releasing single-cell propagules. For multi-species groups we find various optimal strategies. With migration between groups, single-cell propagules are favored. Without migration, larger propagules sizes are optimal; in this case, group-size dependent fissioning rates can prevent the accumulation of cheaters. Our work shows that multi-species groups can evolve reproductive strategies that allow them to maintain cooperative interactions.

## Author summary

In order to reproduce, multicellular organisms and colonial bacteria fragment into offspring groups. Fragmentation modes in nature are very diverse. For instance, some organisms split into two halves, while others release single-celled propagules. These fragmentation modes can have fitness consequences (e.g. small propagules reduce the spread of deleterious mutants). However, the consequences of different fragmentation modes are not yet well understood for groups of cells containing several metabolically interdependent species, such as complex biofilms. We developed a multilevel selection model to investigate the effect of fragmentation mode on the accumulation of deleterious mutants when groups contain multiple species. In such groups, small propagules are not always a viable strategy, because they may harbor low species diversity. We find that alternative mechanisms, such as migration of cells between groups and group-size dependent fissioning rates, can prevent the accumulation of mutants. We also find that multilevel selection can lead to the evolution of fragmentation strategies that allow multi-species groups to thrive in the face of deleterious mutations.

## 1 Introduction

Reproduction is a fundamental feature of life and the *sine qua non* of Darwinian evolution [1–3]. Despite its centrality in natural selection, there appears to be no unique optimum approach to reproduction in multicellular organisms [4–7]. Whenever individual cells abandoned solitary life to form groups—ranging from loose collectives [8] to colonial and multicellular organisms—they came up with a surprisingly diverse menagerie of strategies for the production of reproductive propagues [3].

Many multicellular eukaryotes reproduce by undergoing single-cell bottlenecks. For example, sexually reproducing organisms produce unicellular gametes. Single-cell bottlenecks are also common in plants and animals that reproduce asexually, such as the Amazon molly *Poecilia formosa* [9], several weevils of the Curculionidae family [10], and many angiosperms predominantly in the Asteraceae, Rosaceae, and Poaceae families [11]. An alternative to single-celled propagules is vegetative reproduction in which the offspring develops from a multicellular propagule. This type of reproduction may involve specialized structures, such as conidia (in fungi) or gemmae (in algae, mosses and ferns) [12], or it may happen simply by budding (e.g., in hydra [13]) or by fission (as in some flatworms [14]).

This wide variation in modes of fragmentation is not limited to eukaryotes. Some bacterial aggregations, such as the clusters formed by *Staphylococcus aureus*, reproduce by releasing single-celled propagules [15]. Others, such as filamentous cyanobacteria, reproduce vegetatively: dividing cells remain physically connected, and fragmentation of these aggregates creates new chains [16]. A single parent individual may also divide into two equally-sized multicellular offspring. This occurs, for instance, in the multicellular collectives formed by magnetotactic bacteria [17]. Alternatively, one parent may simultaneously give rise to many equally-sized offspring. The 16-celled colonial alga *Gonium pectorale* takes this strategy to the extreme, by dispersing into 16 individual cells [18].

Why is there such wide variation in the number and size of reproductive propagules? A research program initiated by Kondrashov [6] attempted to answer this question by considering the evolutionary advantages of unicellular propagules relative to vegetative propagules. When offspring develop from a single-cell propagule, they are genetically homogeneous. If the propagule carried any deleterious mutation, its phenotypic effects will not be masked or compensated by wild-type cells. Therefore, reproductive bottlenecks ensure that natural selection is more efficient at eliminating deleterious mutations [6, 7, 19, 20]. Normally, in the absence of genetic recombination, deleterious mutations accumulate in the population in a process known as Muller’s ratchet [21], becoming abundant and decreasing the population’s mean fitness. This decrease in fitness is called mutation load [22–24]. In extreme cases, the resulting maladaptation can lead to severe declines in population size, accelerating the accumulation of deleterious mutations by genetic drift. This positive feedback, which may lead to extinction, is termed mutational meltdown [25]. By facilitating the purging of deleterious mutations, reproductive bottlenecks slow down the detrimental effects of Muller’s ratchet [20] and reduce mutation load [6, 7].

This family of models also informs our understanding of the evolutionary transition [26] from unicellular life to multicellularity. That is because mutations that are deleterious for the group may be beneficial for the mutant cell itself. Mutations of this type (which Roze, Michod [7] call “selfish mutations”) result in “cheater” cells whose fast growth comes at a cost to their group; they include, for example, cancer cells [27]. Selfish mutations (and cheaters more generally) instantiate a conflict between the direction of selection at the level of the cell and at the level of the group. Reproductive bottlenecks resolve this conflict by reducing genetic variation among cells within offspring, and distributing the variation among progeny groups. This reorganization of genetic variation makes selection at the level of the group more effective than selection at the level of the individual cell, thus furthering the evolutionary transition to multicellularity [7].

While this research program has been fruitful and insightful, it considers only a limited set of propagule production strategies (viz., the production of a propagule of varying size). Recent research by Pichugin, Traulsen, and collaborators [5, 28, 29] has started to address the wide variety of strategies that exist in nature. Their models exhaustively analyse the fitness consequences of every mathematically possible partition of multicellular groups.

All of the models described above focus on single-species groups, such as multicellular or colonial organisms. However, multispecies communities also undergo fissioning and fragmentation. Such communities are particularly common in the microbial world: the majority of microorganisms belong for at least part of their life to multispecies groups, such as mixed biofilms that comprise up to thousands of different species [30]. Just like single-species collectives, multispecies microbial communities display a wide variety of modes of fragmentation. Host-associated communities can be vertically transmitted between host generations or they can be recruited from the environment (both of which are exemplified in insect microbiomes [31]). They can also be formed by a combination of both, which is the case, for example, for the human microbiome [32]. Environmental communities can also form by recruitment (e.g., colonization of marine-snow particles [33]) or from multicellular fragments which detach from mature communities (e.g., some bacterial biofilms [8]).

Even though natural biofilms are heterogeneous communities of different microbial species, organisms within them are well adapted to group life. In mixed biofilms, individuals of different species communicate with each other via quorum sensing (e.g. [34, 35]) and engage in cooperative interactions with each other [30]. Such interactions include cross-feeding, in which byproducts from nutrients that are metabolized by one species then serve as food source for a second species (reviewed in [36]). Cross-feeding is so prevalent that many microbes have lost the ability to synthesize essential metabolites and became metabolically interdependent [37]. This type of metabolic specialization can increase the growth rates of all member species [38–40], and can allow communities to perform otherwise incompatible tasks, such as photosynthesis and nitrogen fixation [41–43]. However, cross-feeding communities are vulnerable to invasion by non-cooperative “cheater” genotypes that take up resources without reciprocating, raising the question of whether cross-feeding communities are evolutionary stable [44–47].

Much like selfish mutations in multicellular organisms, cheaters in mixed biofilms are manifestations of a conflict between levels of selection. In recent years a number of multilevel selection models have been developed to investigate how this conflict can be resolved [48–51]. It was found that stochastic effects and non-equilibrium dynamics can maintain cooperative interactions over evolutionary time, in principle [52, 53]. However, whether cooperative interactions can be maintained critically depends on details of the group level dynamics, such as the timing and mode of group fragmentation. One limitation of these previous studies is that many only investigated a single, or a small number, of different fragmentation modes, impeding a direct comparison of how the fragmentation mode affects the maintenance of cooperative interactions. Gao, Traulsen, Pichugin [54] have started to address this knowledge gap by investigating exhaustively how all possible modes of reproduction can maintain cooperative interactions in a two-genotype community. They found that cooperative interactions are compatible with the fragmentation of a group into either two multicellular halves or multiple independent cells. Although this study is very insightful, the analytical approach used is not trivial to extend to large groups consisting of many interacting species. Hence, we here developed a complementary framework to investigate how the fragmentation mode affects the maintenance of cooperative interactions in multispecies groups of arbitrary size and complexity.

**Fig 1:**
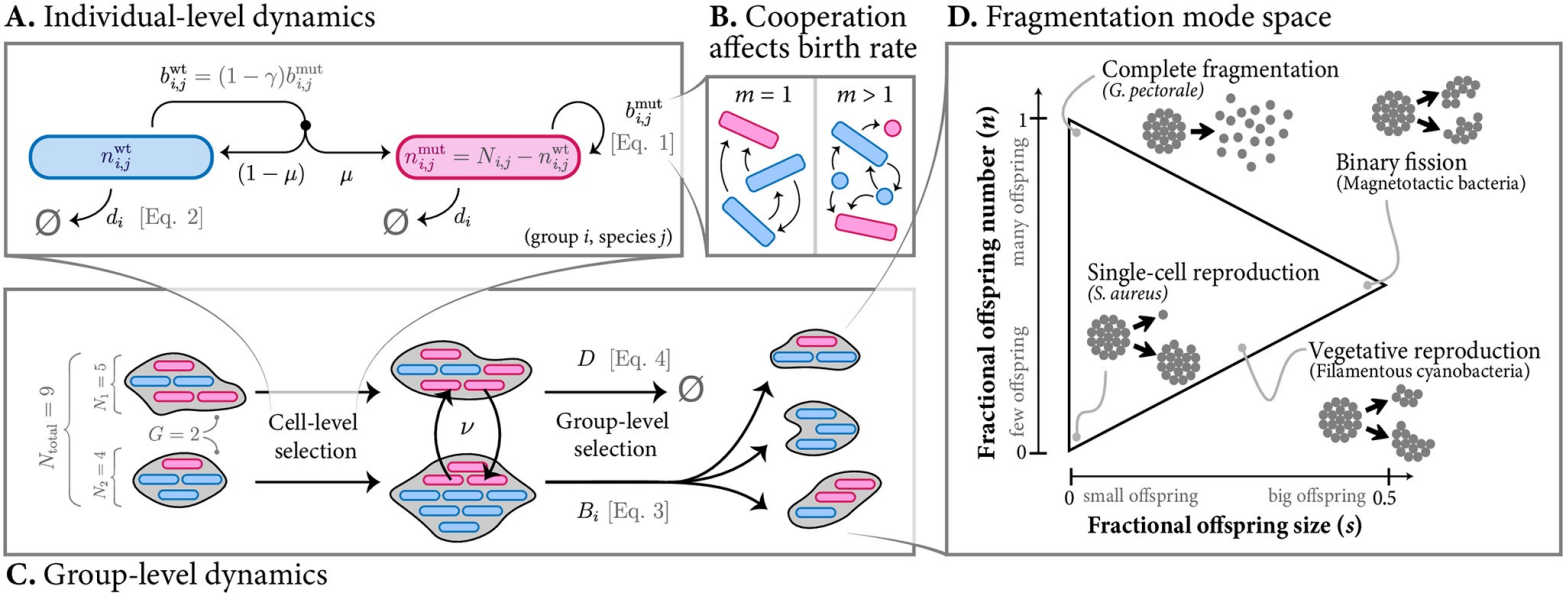
Illustration of the main model ingredients. We consider a community consisting of a varying number *G* of multicellular groups. Dynamics unfolds via simultaneous stochastic birth-death processes at both the individual level (**A**) and the group level (**C**). **A**: Each group *i* is composed of *N*_*i*_ cells, of which *N*_*i*,*j*_ cells belong to species *j*. Of these, a number $n_{i,j}^{\text{wt}}$ are wild-type (blue), and the remaining $n_{i,j}^{\text{mut}}$ are mutants (red). The birth rate of mutants is given by $b_{i,j}^{\text{mut}}$ (Eq 1); the birth rate of wild-type cells is $b_{i,j}^{\text{wt}}=\left(1-\gamma \right)b_{i,j}^{\text{mut}}$, where *γ* is the cost of cooperation. Mutations occur at birth with a probability *μ*. All cells in the group have the same death rate, *d*_*i*_ (Eq 2). **B**: Cooperation on the part of wild-type cells (e.g., by producing public goods—indicated with arrows—that are consumed by other cells) determines cell birth rate. In the single-species case (*m* = 1) cooperation occurs between individuals of the same species. In the multi-species case (*m* > 1) cooperation occurs across species (e.g., cross-feeding); species are denoted by differently-shaped icons. **C**: Groups in the community also undergo a birth-death process. (Group-level and individual-level dynamics unfold simultaneously, but they are depicted sequentially for simplicity.) Group death (termed ‘extinction’) occurs at a rate *D* (Eq 4), and group birth (termed ‘fission’ or ‘fragmentation’) occurs at a rate *B*_*i*_ (Eq 3). At a rate *ν*, cells can also migrate between groups. **D**: When a fission event occurs, the parent group fragments into offspring groups. The number and size of offspring groups is determined by the group’s fragmentation strategy. All possible fragmentation strategies in our model can be described using a two-dimensional phase space. Two parameters (*s* and *n*) determine the expected size and number of offspring, respectively (as a fraction of the number of cells in the parent group). Cartoons exemplify some modes of fragmentation. We refer to the three corners of the triangle (complete fragmentation, single-cell reproduction, and binary fission) as archetypal modes of fragmentation.

We developed an individual-based, multilevel selection [55, 56] model of groups consisting of cells that interact mutualistically (Fig 1). Cells in the group stochastically give birth or die (Fig 1A), while groups stochastically go extinct or reproduce (Fig 1C). Whenever a group reproduces, it follows a predefined “fragmentation mode” that describes how its constitutive cells are arranged over the offspring groups (Fig 1D). Both the size and the number of offspring groups can vary continuously allowing us to fully investigate how the mode of fragmentation affects the ability of the group to resist mutations.

Drawing from the lessons of models on single-species collectives, we expect that in multi-species groups different fragmentation modes could have strong implications on how well the system can cope with mutants. For example, by decreasing variation within groups, we expect that reproductive bottlenecks intensify group selection and help purge cheater mutants. On the other hand, we expect that the benefits of strong bottlenecks diminish in complex multi-species communities, because small propagules are less likely to contain all of the species that are metabolically interdependent.

Our model confirms these expectations: we find that high-diversity communities face a reproductive trade-off: tight bottlenecks result in the elimination of deleterious mutations, but they also decrease species diversity; the opposite is true when offspring groups are large. Using our model we also investigated strategies that can relieve this trade-off: adding migration between groups reconciles single-cell bottlenecks with the need for diversity, while making group fragmentation rate dependent on group size increases the strength of group level selection thus reducing mutant load. Finally, we extend our model to investigate how group fragmentation rates evolve and we show that groups always evolve to the fragmentation mode that maximizes their resistance to mutants.

## 2 Model

Previous work has shown that stochastic effects and non-equilibrium dynamics can be essential in maintaining cooperative interactions in multi-species groups [52, 55, 56]. We therefore developed a stochastic, individual-based model, to investigate how the fragmentation mode affects the ecological and evolutionary stability of multi-species cross-feeding groups. Our model builds on a previously developed multi-level selection framework [55, 56] and explicitly considers the dynamics at two levels: the individual (i.e. cell) and the group level (Fig 1). On both levels we model the dynamics using a birth-death process; to prevent confusion we use the terms fission and extinction for group-level birth and death events, respectively. We consider communities consisting of *m* different species, that interact via cross-feeding interactions. Individuals of each species can be of two types: wild-type (cooperator), or mutant (cheater); the latter use resources produced by the wild-type cells but do not reciprocate in producing them, and as result they grow faster than wild-type cells. The numbers of individuals and groups are discrete and vary stochastically as a result of birth/death and fission/extinction events, respectively. Throughout, we will assume the simplest possible functions to describe the rates at which these events happen, with the constraints that the rate functions are biologically plausible and computationally feasible (i.e., such that population sizes remain bounded); see Fig A in S1 Text for an illustration of the rate functions. Our framework is fully compatible with more complex (e.g. nonlinear or state-dependent) rate functions, however we leave the exploration of these more complex effects for future work.

**Notation**. We consider a community consisting of a stochastically varying number *G* of multicellular groups. Within each group *i*, there are *N*_*i*_ individual cells, which may belong to any of *m* different species. Regardless of species, each cell is either wild-type (cooperator) or mutant (cheater). We denote the number of cells of species *j* ∈ {1, …, *m*} within group *i* as *N*_*i*,*j*_, so that $N_{i}=\sum_{j=1}^{m}N_{i,j}$. Of these, a number $n_{i,j}^{\text{wt}}$ are wild-type and the remaining $n_{i,j}^{\text{mut}}=N_{i,j}-n_{i,j}^{\text{wt}}$ are mutants.

The number of cells within each group changes due to cell birth and cell death events, which occur at rates *b*_*i*,*j*_ and *d*_*i*_, respectively (section 2.1). Simultaneously, the groups themselves undergo fission (group birth) and extinction (group death) events, at rates *B*_*i*_ and *D*, respectively (section 2.2). Finally, migration of cells between groups occurs at a rate *ν*.

### 2.1 Cell dynamics

**Cell birth rate**. A cell’s birth rate depends on cooperative interactions with other cells in the same group. At a cost *γ* to themselves, wild-type cells (cooperators) contribute to these interactions, increasing the birth rate of others in the group. However, mutations turn cooperators into cheaters, who reap the benefits of interactions without contributing (we ignore back-mutation). As a result, mutants always multiply faster than conspecific wild-type cells in the same group, but their presence slows the overall growth of the group (Fig 1A).

We consider two possibilities: single-species systems (*m* = 1), where groups resemble multicellular organisms, and multispecies systems (*m* > 1), where groups are akin to mixed biofilms. When *m* = 1, cooperation occurs between individuals of the same species, whereas for *m* > 1, cooperation occurs across species, thus resembling obligate mutualistic interactions (Fig 1B). Then, the per capita realized birth rate of a mutant cell is equal to

$$b_{i,j}^{\text{mut}}=\{\begin{array}{ll} \frac{n_{i,j}^{\text{wt}}}{N_{i}}, & \text{if}\;m=1,\\ m^{\left(m-1\right)}\prod_{k\neq j}^{m}\frac{n_{i,k}^{\text{wt}}}{N_{i}}, & \text{otherwise}, \end{array}$$ (1)

where the term *m*^(*m*−1)^ ensures that the total number of cells at equilibrium is the same across different values of *m* (note that this term does not affect the results, as we never compare population densities between different values of *m*). The second term of Eq 1 describes the production, by each individual, of a fixed amount of public good, which is then taken up by all cells in the group. Growth is limited by the public goods produced by other group members: when *m* = 1, a cell can only grow in the presence of conspecific cooperating partners, and when *m* > 1 a cell of species *j* can only grow in the presence of cooperating partners of all *m* − 1 other species ($n_{k,i}^{\text{wt}}>0$ for all *k* ≠ *j*). Note that, when *m* > 1, growth does not depend on the presence of conspecifics, because it is meant to represent mutualistic interactions such as cross-feeding. The realized birth rate of a wild-type cell is then $b_{i,j}^{\text{wt}}=\left(1-\gamma \right)\cdot b_{i,j}^{\text{mut}}$, where *γ* is the cost of cooperation.

Whenever a new cell is born, it stays in the same group as the parent. If a wild-type cell gives birth, with probability *μ* it generates a mutant cell of the same species and with probability (1 − *μ*) the offspring remains wild-type. We only consider mutations from wild-type to mutant in same species: there is no back-mutation or mutations across species.

**Cell death rate**. We assume that the per capita death rate *d*_*i*_ for all cells in group *i* increases linearly with the total population size in the group (i.e. logistic growth):

$$\begin{array}{c} d_{i}=\frac{N_{i}}{K_{\text{cells}}}, \end{array}$$ (2)

where *K*_cells_ is the within-group carrying capacity. This assumption is needed for computational reasons, to keep group size bounded, but it is also biologically plausible: even in the presence of cooperative cross-feeding interactions, cells will often compete for other limiting resources (e.g., space, other nutrients), giving rise to density dependence. In S3 Text we consider an alternative approach where the death rate is constant (*d* = 1/*K*_cells_) and where we instead keep group size bounded by forcing groups to fission when they reach a size of *K*_cells_ individuals.

### 2.2 Group dynamics

**Group fission rate**. We use the term “fission rate” to refer to the rate at which groups reproduce. All else being equal, groups with higher fission rates are favored by group selection. As a baseline, we assume that groups fission with a constant rate *B*_0_. To be able to change the strength of group selection in our model, we additionally explore the possibility that the fission rate depends on the properties of the group. We focus on the simplest possible dependence, where the fission rate increases linearly (with constant of proportionality *σ*) with the size of the group *N*_*i*_ (normalized by the carrying capacity of the group). In summary,

$$\begin{array}{c} B_{i}=B_{0}+\sigma \cdot \frac{N_{i}}{K_{\text{cells}}}. \end{array}$$ (3)

**Fragmentation modes**. When a group of size *N*_*i*_ reproduces, it fissions into a parental group and one or more offspring groups, according to its mode of fragmentation. We assume that the offspring size is homogeneous, i.e., all offspring groups have the same expected size (this reduces the space of possible modes of fragmentation when compared to the more exhaustive approach used by [5]). As both group and offspring size are discrete numbers, in practice offspring group size cannot be perfectly homogeneous, see below for details. Our model accommodates a wide variety of modes, without attempting to be exhaustive. To do this, we consider a triangular space of fragmentation strategies that is defined by two parameters (Fig 1D).

The first parameter (0 < *n* ≤ 1) determines the expected *number* of cells that are transmitted to offspring, as a fraction of the parent’s cell number. Whenever a group reproduces, we first draw this number of cells, *N*_offspring_, from a normalized Poisson distribution with expectation *n* ⋅ *N*_*i*_ and support on {1, …, *N*_*i*_}. The second parameter (0 < *s* ≤ 0.5) determines the expected *size* of each offspring group, again as a fraction of the parent’s cell number. This size, *S*_offspring_, is drawn from a normalized Poisson distribution with expectation *s* ⋅ *N*_*i*_ and support on {1, …, *N*_offspring_}. From these two quantities we then calculate the total number of offspring groups, *G*_offspring_ = ceil(*N*_offspring_/*S*_offspring_). The first *G*_offspring_ − 1 offspring groups are all assigned *S*_offspring_ cells, which are randomly sampled (without replacement) from the parent group. The final offspring group is assigned the remaining cells, again by random sampling (without replacement) from the parent group. Finally, we remove all cells that are transmitted to the offspring groups from the parent group. The parameter *s* is analogous to the propagule size parameter in Kondrashov [6] and Roze, Michod [7]. The only valid combinations of parameters obey the condition *s* < *n* < 1 − *s*. The lower triangle is excluded because it is logically impossible, whereas the upper triangle is excluded because one of the groups that result from the fissioning process is arbitrarily labelled as the parent group. For example, imagine a group of size 10 fragments into five groups of size 2. We label one of these groups as the parent groups, resulting in four offspring groups of size 2 plus a parent group of size 2 (i.e., a point on the upper diagonal).

We refer to the three corners of this parameter space as *archetypal* fragmentation modes (Fig 1D): in *single-cell reproduction*, the parent group produces a single offspring group by releasing a unicellular propagule; in *complete fragmentation*, the entire parent group disperses into its constituent cells; finally, in *binary fission*, the parent group divides into two approximately equally-sized groups (when parent group size is odd-numbered, the two offspring groups will differ in size by one cell). Every mode of fragmentation described in section 1 can be accommodated in this parameter space.

**Group extinction rate**. We use the term “extinction rate” to refer to the rate at which groups die. We assume that the extinction rate increases linearly with the total number of cells in the entire community *N*_total_ = ∑_*i*_ *N*_*i*_, resulting in the per group extinction rate

$$\begin{array}{c} D=\frac{N_{\text{total}}}{K_{\text{total}}}, \end{array}$$ (4)

where the parameter *K*_total_ roughly scales the total number of cells in the community. This assumption ensures that the total population size remains bounded. Biologically this corresponds to the scenario where all cells, independent of in which group they are located, compete for the same limiting resource (e.g. space or a nutrient). In the S3 Text we explore some alternative forms of group-level density dependence: one situation where the group extinction rate increases linearly with the number of groups, and another where the group extinction rate decreases with group size (in addition to increasing with number of cells). For these scenarios we obtain qualitatively similar results (Figs C and D in S3 Text).

**Migration rate**. Cells can migrate between groups; we consider the simplest possible form of unstructured migration, where cells leave their own group at a constant per capita migration rate *ν* and join a second, randomly chosen group.

### 2.3 Evolution of the fragmentation mode

So far we have considered the fragmentation mode of a group as a fixed strategy. However, the fragmentation mode of a group is likely determined by traits of the cells that make up the group (e.g. the stickiness of the extra-cellular matrix that binds cells). As a result, the fragmentation mode is potentially an evolvable trait. To study the multilevel evolutionary dynamics of fragmentation mode, we characterize every cell in the population with a quantitative phenotype vector (*s*, *n*). These traits could represent, for example, the properties of the materials that are excreted into the extracellular matrix. The average trait value of the group determines the group’s position in state space (Fig 1D) and hence its mode of fragmentation. Thus, the properties of the individual cells give rise to an emergent group property. When cells reproduce, their offspring inherit their parent’s trait values; however, with a small probability *μ*_*s*_ or *μ*_*n*_, small-effect mutations may occur. For computational efficiency, we discretize the phenotype space and assume that mutations always occur between adjacent phenotype bins. We do not allow mutants outside the bounds of the phenotype space: any such mutants are moved along the *n*-direction and placed on the boundary of the allowed space.

### 2.4 Model implementation

The individual-based simulations were implemented using a Gillespie algorithm [57]. The code was implemented in Python [58] using the NumPy package [59]; Numba [60] was used to accelerate the computations. Simulation data was processed and visualized with R [61], using the tidyverse meta-package [62] as well as the following packages: cowplot [63], egg [64], ggpubr [65], glue [66], here [67], patchwork [68], and scales [69]. Interoperability between Python and R was provided by the reticulate [70] package. The code and data files required to reproduce the results and the figures are available at Zenodo [71]: http://doi.org/10.5281/zenodo.5102670.

All simulations were started with 100 groups, of equal size and composition (within rounding errors). The initial groups consist of *K*_cells_/2 wild-type cells (i.e., 50 cells at default parameters), split equally over the *m* species (within rounding errors). The state of the community was sampled every time-unit (in our model parameterization, the maximum birth rate of cells is 1, time is thus measured in units of the shortest possible generation time of cells). To reduce the effect of stochastic fluctuations we averaged all model outputs over a moving window of 200 time-points. Simulations were run until the community reached a steady-state (equilibrium). This criterion was evaluated automatically by calculating the magnitude of the fluctuations (defined as the root-mean-square error over the last 200 time-points) in the moving-average values of the total number of wild-type cells and the total number of groups in the community. Steady state was defined as the time where the total number of wild-type cells fluctuated by less than 1% and the total number of groups by less than 5% compared to their time-averaged values. These thresholds were determined based on visual inspection of the trajectories of many individual simulations sampled over the full parameter space of our model. In addition, simulations were stopped when the community went extinct, when the population size grew without bounds, or when the required computational time grew excessively large. In these last two cases, the model outputs where excluded from further analysis (this explains the missing data in parts of the parameter space in some of the figures). To calculate the maximum mutation rate each strategy could withstand before undergoing mutational meltdown, we performed simulations as described above. We started simulations at the highest possible mutation rate (*μ* = 1), and progressively lowered the mutation rate until the community reached a non-zero steady state. For the evolution runs, we initiated the simulation with a homogeneous populations (i.e. all cells having identical (*s*, *n*) traits). We then let the simulations run for one million time-units, sampling the state of the population every thousand time-units.

## 3 Results

### 3.1 Fragmentation in single-species groups

#### 3.1.1 Multilevel selection can maintain cooperators and avert mutational meltdown

**Fig 2:**
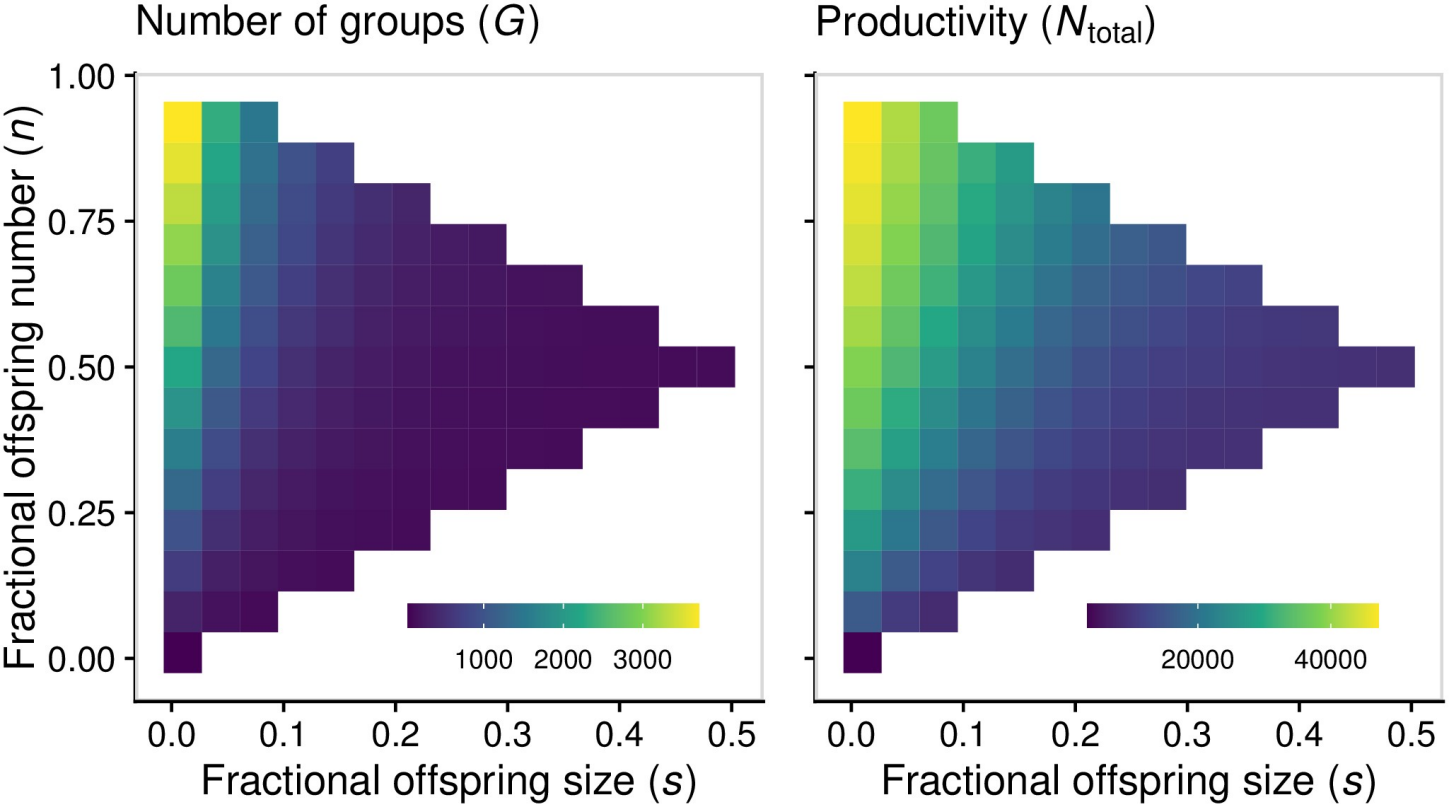
In the absence of mutations, with a single species, complete fragmentation into single cells maximizes equilibrium population size, measured either as total number of groups (*G*) or as total productivity (*N*_total_). Parameters: *μ* = 0; all other parameters set to the default values (Table A in S1 Text).

We will first consider groups consisting of a single species. When there are no mutations, the strategy of complete fragmentation maximizes equilibrium population size, measured either as total number of groups (Fig 2A) or as total number of cells (i.e., productivity, Fig 2B). This can be explained based on our choice of a density-dependent death rate: the steady-state distribution of group sizes depends on the fragmentation mode (Fig B in S2 Text). Complete fragmentation keeps group size small and as a result the average death rate is lower compared to other fragmentation modes where groups can grow larger. When the death rate is constant (no-density dependence) the productivity is similar for all fragmentation modes, though the number of groups is still maximized for complete fragmentation (Fig E in S3 Text). Density dependence thus has a rather strong effect on how the productivity depends on the fragmentation mode, however we show in S3 Text that all our other conclusions (e.g., with regards to the effect of mutations or species diversity) are robust to whether the cell death rate is density dependent or not (Fig F in S3 Text).

**Fig 3:**
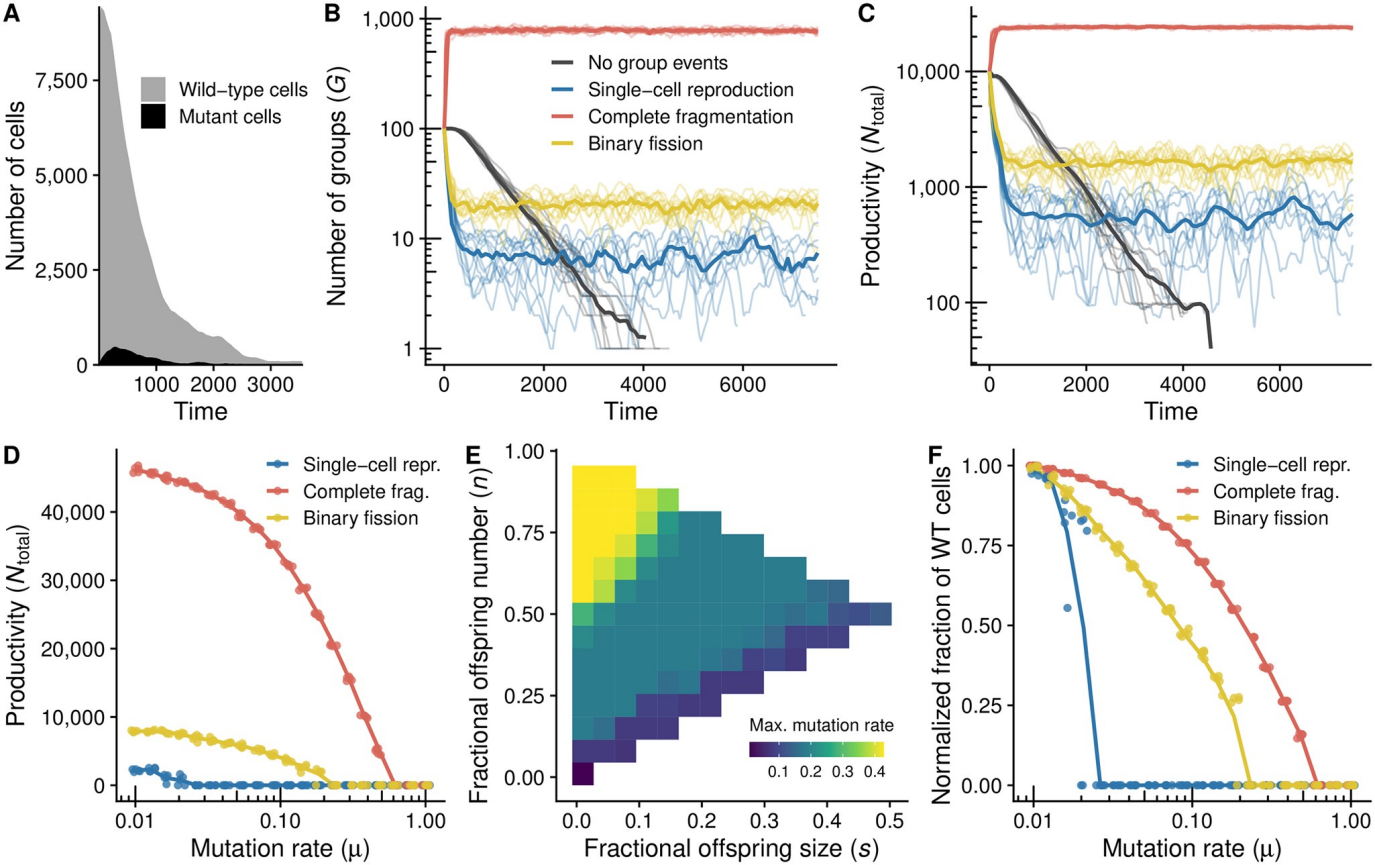
When there are no group events, mutations cause community extinction, but group-level events prevent this fate, as exemplified by the three archetypal modes of fragmentation. The figures show the time-dynamics of populations (initiated with 100 groups, each containing 100 cooperator cells). **A**: Example replicate with no group events (i.e., no group fission and extinction), showing fast initial rise in the number of mutants and the consequential decrease in the total cell number, resulting in extinction. **B, C**: Temporal dynamics of the number of groups (**B**) and total productivity (**C**) for different reproduction modes. Thin lines are moving averages of individual replicates; solid lines are averages across replicates. **D**: By simulating population dynamics for various values of the mutation rate (*μ*), we can identify the value at which the population undergoes mutational meltdown–driven extinction (i.e., the *maximum mutation rate* at which the population can persist). **E**: For every point in the strategy space, we calculated the maximum mutation rate (shown in panel **D** for the three archetypes). **F**: The percentage of wild-type cells at equilibrium decreases with mutation rate (*μ*); the plot depicts the fraction of wild-type cells relative to the equilibrium fraction when *μ* is very small (*μ* = 0.01). Parameters: for the lines with group events, *B*_0_ = 0.01; all other parameters set to the default values (Table A in S1 Text).

Above we considered groups consisting of wild-type cells only, but how does the presence of cheater cells affects the dynamics of single species groups? Because mutations constantly create cheaters, we expect slower growing wild-type cells to go extinct over evolutionary time in the absence of group-level events. Furthermore, because all cells depend on the presence of cooperators to reproduce, this process is expected to lead to mutational meltdown: the extinction of the entire community due to the influx and spread of mutations. Indeed, when we simulate populations initially containing no mutants and set group-level rates to zero (i.e., no group fissions or extinctions), we observe the accumulation of mutations, leading to the decrease in group size and eventual extinction of the community (Fig 3A and black lines in Fig 3B and 3C).

However, group level selection can maintain cooperators and prevent meltdown. Cells in groups with many cooperators multiply faster than cells in groups with few cooperators. Furthermore, when these groups fission, they give birth to offspring groups that also have higher frequency of cooperators. Bigger groups are also less likely to collapse due to the stochastic death of all its member cells (driven by the accumulation of mutations and by genetic drift), and will therefore remain in the population for longer and have more opportunities to fission. Accordingly, when group-level rates are nonzero, selection at the level of the group can avert mutational meltdown (Fig 3B and 3C).

#### 3.1.2 Complete fragmentation minimizes mutation load

Previous theoretical work [6, 7, 19, 20] predicts that, in single-species systems, small offspring sizes should be most resilient against mutational meltdown, since single-cell bottlenecks expose harmful mutations to natural selection. In agreement with these predictions, we found that the mode of group fragmentation has important consequences for the capacity of the population to persist in the presence of mutations (Fig 3D). In single-species communities (*m* = 1), the complete fragmentation archetype and strategies close to it are able to avoid mutational meltdown-driven extinction even for relatively high rates of mutation when compared to other strategies (Fig 3D and 3E). This is because, although the average frequency of wild-type cells per group always decreases with mutation rate, the relative decrease is smaller for this archetype (Fig 3F), as would be expected given the role of tight bottlenecks in eliminating deleterious mutations.

### 3.2 Fragmentation in multispecies groups

#### 3.2.1 Multispecies communities are more vulnerable to mutational meltdown

**Fig 4:**
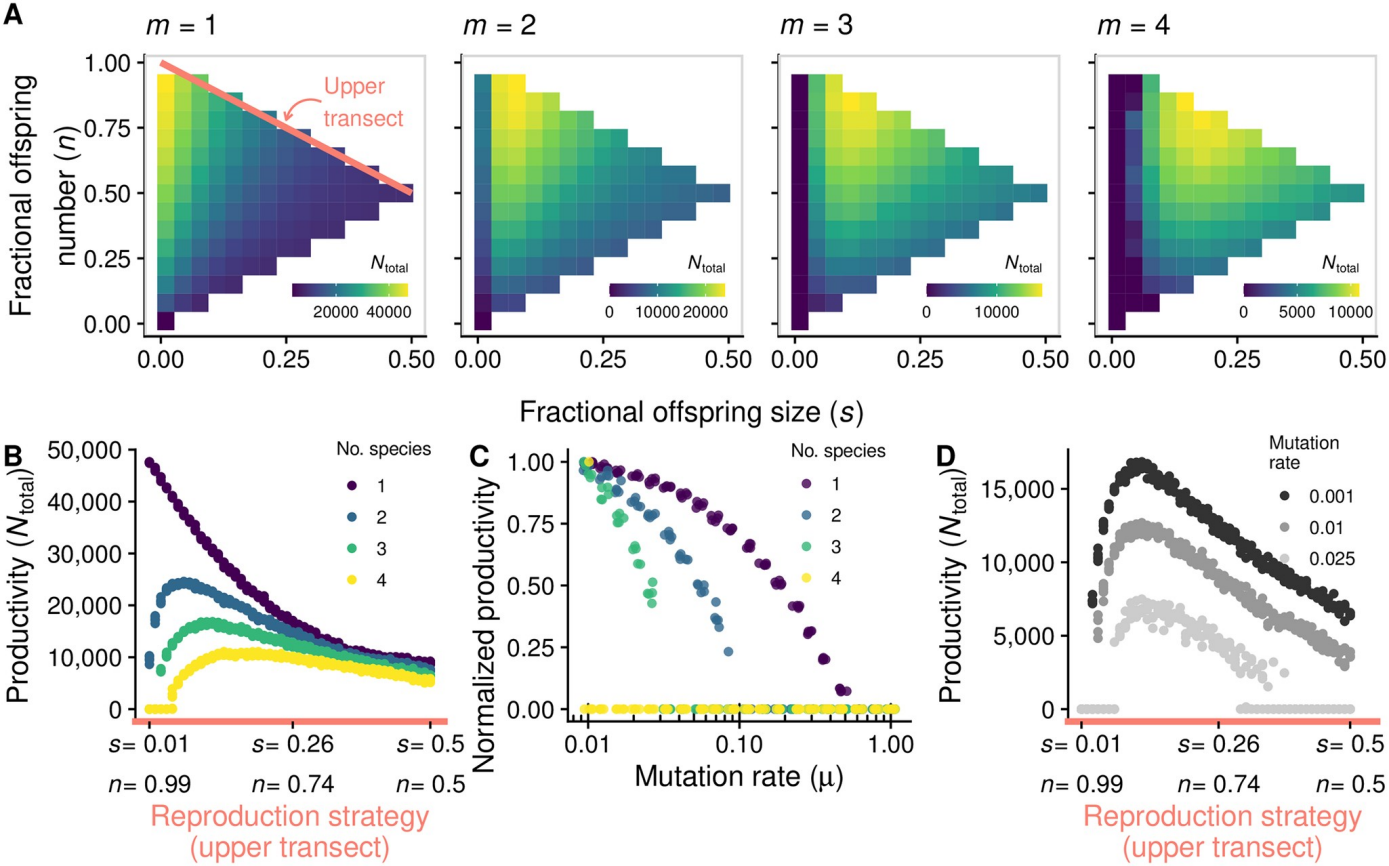
When community complexity is high, the productivity peak shifts away from unicellular bottlenecks. **A**: The color indicates equilibrium community productivity (*N*_total_). As the number of species (*m*) increases, the strategy that maximizes *N*_total_ moves rightward along the upper transect (pink line) of the strategy space. **B**: Equilibrium productivity as a function of strategies along the upper transect of the strategy space (corresponding to the pink line from panel **A**; ranges from complete fragmentation, on the left of the *x* axis, to binary fission, on the right), for different numbers of species. **C**: Equilibrium productivity decreases with mutation rate (*μ*); this decrease is faster for higher number of species (exemplified here for *s* = 0.1, *n* = 0.9.). The plot depicts the productivity at equilibrium relative to the case in which *μ* is very small (*μ* = 0.01). **D**: Some strategies that do well with small *μ* (large offspring) are not viable when *μ* is large (shown here with *m* = 3). Parameters: all parameters are set to the default values, unless otherwise indicated (Table A in S1 Text).

We have seen that, when single-species groups reproduce by complete fragmentation, they are most resistant to mutational meltdown. Multispecies communities, in contrast, cannot resort to complete fragmentation, since in small fragments there is a high chance that one or more heterospecific types are missing. In the extreme case of unicellular fragments, the offspring cell can never grow because of the absence of mutualistic interactions. In the absence of mutations, multispecies communities are thus most productive when groups have larger offspring, which allows offspring to maintain a variety of species. In other words, for increasing species number, the productivity maximum moves rightward along the upper diagonal of the phenotype space (Fig 4A and 4B).

Because multispecies communities need larger offspring, they are also more vulnerable to mutants: the higher the number of species, the faster the community productivity goes down with increasing mutation rates (Fig 4C). Therefore, there is a trade-off between resistance to mutational meltdown (low offspring size) and the maintenance of mutualistic interactions (high offspring size, Fig 4D).

#### 3.2.2 Size-dependent fragmentation rate and migration prevent mutational meltdown in multispecies communities

We have seen that (consistent with previous studies) multicellular organisms can reduce mutation load and prevent mutational meltdown by reducing propagule size (section 3.1.2), but that this strategy is not available in multispecies communities, since it deprives cells in daughter groups of their mutualistic partners (section 3.2.1). What reproductive strategies, then, allow for more complex multispecies communities to resolve this trade-off?

**Fig 5:**
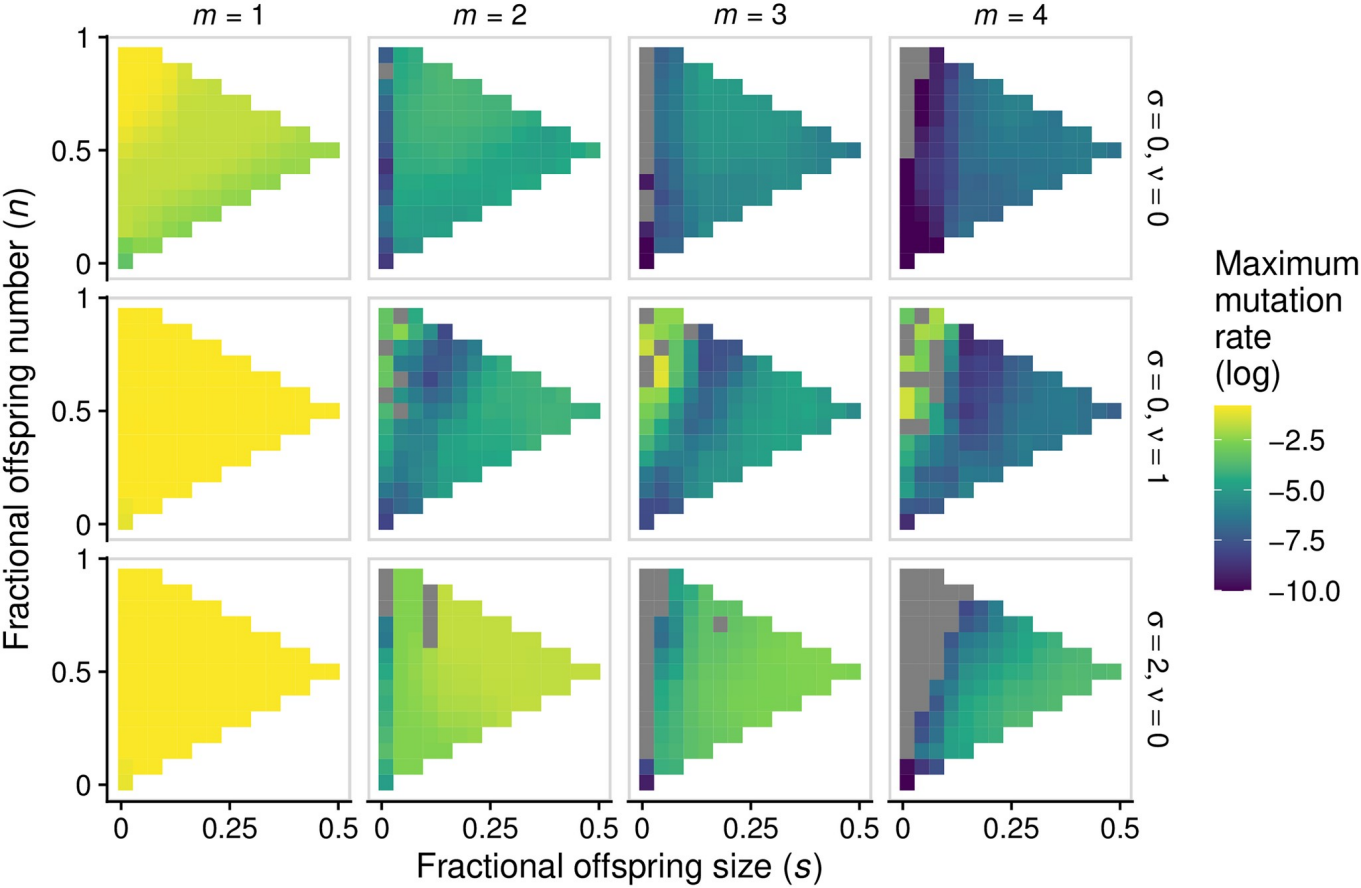
Migration and size-dependent fragmentation rate allow multispecies communities to resist mutational meltdown. Each panel shows, for each position in the strategy space, the maximum mutation rate a population can experience before going extinct (similar to Fig 3E). Columns (from left to right) depict increasing number of species. Rows depict: no size-dependent fragmentation and no migration (*top*); migration and no size-dependent fragmentation (*center*); size-dependent fragmentation and no migration (*bottom*). Grey squares correspond to communities for which the maximum mutation rate is outside the range of our simulations or numerical errors. For each value of mutation rate, we assessed ten replicates per fragmentation mode; we then calculated the mean (across replicates) of the logarithm of maximum mutation rate. Each point in the figure corresponds to a nearest-neighbour average of this quantity. Parameters: all parameters are set to the default values, unless otherwise indicated (Table A in S1 Text). Figs G and H in S4 Text comprise a wider range of values of *σ* and *ν* and depict raw values of maximum mutation rate (rather than nearest-neighbour averaged values).

One alternative is size-dependent fragmentation rate. Larger groups may be more likely than smaller ones to undergo fission and thus produce offspring groups (in our model, this is achieved by increasing the slope parameter *σ* in Eq 3). Because all groups have equal extinction rate, groups with higher fission rate are favored by group selection. Hence, increased size-dependence in fissioning rate intensifies the effectiveness of group selection in purging mutants, since groups with many mutants grow slower and thus take longer to reproduce. By increasing the importance of group selection relative to individual-level selection, size-dependent fissioning should allow complex communities to withstand high rates of mutation. To test this hypothesis we calculated, for different values of *σ*, the highest mutation rate that communities can withstand, over the entire strategy space. In other words: for each fragmentation mode we found the highest mutation rate that the population can handle before if collapses; we then selected the fragmentation mode for which this mutation rate is highest. We will later see that this strategy is also an evolutionary attractor, so we expect that this is the mode of fragmentation of groups at evolutionary equilibrium. The results confirm our prediction that at higher values of *σ*, communities can survive in the presence of higher mutation rates (Fig 5; see also Fig G in S4 Text for a broader range of parameters).

Another alternative is migration of cells between groups, which is common in bacterial biofilms. In multilevel selection theory, migration is often considered to be detrimental to group selection, because it decreases variance between groups and increases the role of individual-level selection [51, 72–74]. However, in multi-species systems, low to intermediate levels of migration can be beneficial, because they allow small offspring to recruit individuals from the environment and, thus, achieve the diversity necessary for mutualistic interactions (Fig 5; see also Fig H in S4 Text for a broader range of parameters).

Size-dependent fragmentation and migration are thus two possible solutions to the challenge of resisting the accumulation of mutations while also maintaining species diversity within groups. Size-dependent fragmentation increases the strength of group selection, which purges deleterious mutations, thus making small offspring sizes unnecessary. In contrast, migration allows for the recruitment of heterospecific cells, which allows diversity to be maintained even when offspring sizes are small.

### 3.3 Evolution of fragmentation modes

So far we have considered communities where every group has the same fragmentation mode. In this section we consider what happens when groups in a community vary with respect to their reproductive strategy. We have seen that fragmentation mode has implications for quantities such as community productivity (total number of cells), number of groups, and total frequency of mutants in the community. However, different strategies may maximize each of these quantities. For example, when fragmentation rate is size-dependent, the number of groups is maximized when the slope of the fission function (*σ*) is small (close to the complete fragmentation archetype), whereas the total community productivity is maximized when *σ* is large (close to the binary fission archetype). Given that both the number of groups and the number of cells affect selection in different ways at both levels of organization, it is not obvious which mode of fragmentation is favored by natural selection under different conditions.

**Fig 6:**
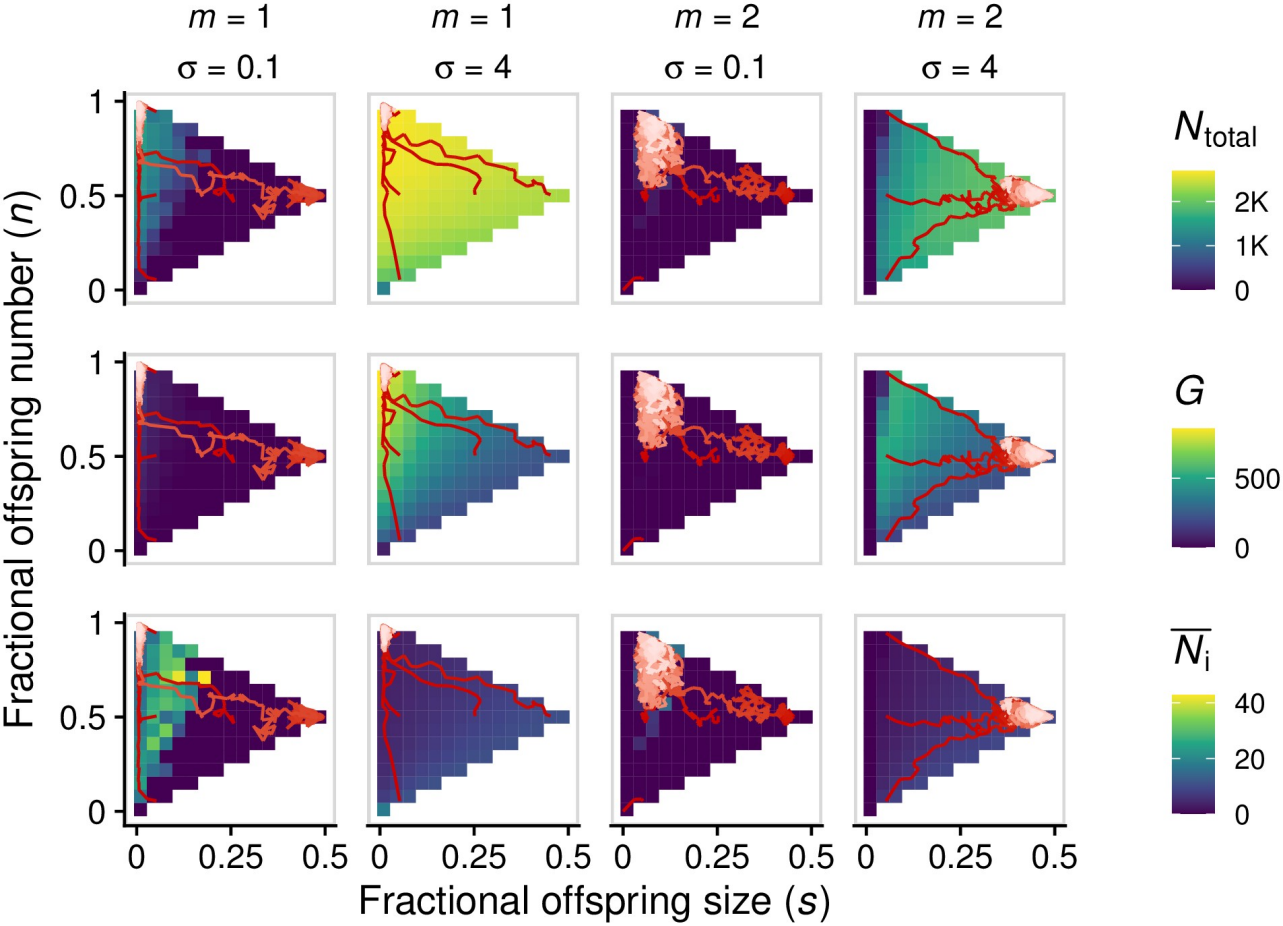
Evolution of fragmentation mode maximizes total number of cells (*N*_total_, top row) rather than other quantities such as number of groups (*G*, middle row) or average group size ($\bar{N_{i}}$, bottom row). Red lines represent evolutionary trajectories of the average mode of fragmentation over time (darker shades of red correspond to earlier time points). For each value of *σ*, we assessed five initial phenotypes. Parameters: all parameters are set to the default values, unless otherwise indicated (Table A in S1 Text), except: *K*_cells_ = 100, *B*_0_ = 0.01, *K*_total_ = 30, 000 (when *σ* = 0) or *K*_total_ = 10, 000 (otherwise), *μ*_*s*_ = *μ*_*n*_ = 10^−2^.

To answer this question, we simulated the multilevel evolutionary dynamics (section 2.3). Across a diverse set of parameters, we found that strategies that maximize community productivity (i.e., total number of cells) are evolutionary attractors and endpoints of evolution (for some examples, see Fig 6). Therefore, when complexity is low, we expect evolution to lead to tight bottlenecks, but for more complex communities with more than one species, we expect group selection to promote the evolution of binary fission. When there are multiple species, the evolutionary outcome will depend on what mechanisms are in place to resolve the trade-off between maintaining complexity and eliminating deleterious mutations; for example, size-dependent fragmentation favours the evolution of binary fission (Fig 6).

## 4 Discussion

Reproduction is the defining characteristic of life, yet organisms across the tree of life reproduce in many different ways. In organisms that have single-celled gametes, there is less within-organism variation than in organisms with many-celled propagules, which increases the strength of natural selection in removing deleterious mutations [6, 7, 19, 20]. Hence, single-celled bottlenecks allow populations to reduce mutation load. In this paper, we studied how this process affects the mode of fragmentation of multispecies collectives of organisms, such as microbial biofilms. We found that complex communities face a reproductive dilemma. On the one hand, their persistence relies on aligning the Darwinian interests of the group and its individual cells, which can be achieved by small reproductive bottlenecks (not unlike single-species organisms). On the other hand, due to stochastic sampling, small daughter groups lack the species diversity that multispecies communities rely on for ecological success. There is a tug-of-war between two competing selection pressures affecting daughter group size: maintaining species diversity while reducing mutation load. We explored two alternative solutions to this dilemma: migration (which makes small groups viable) and size-dependent fragmentation (which reduces load even within large groups).

Migration of cells between groups allows small groups to acquire species diversity. Due to stochastic sampling, some newly born groups are free of mutants, but may also lack mutualistic partners. Thanks to migration, these groups will be able to recruit individuals of other species, whose presence is necessary for cell growth. Mutants also migrate, which may be detrimental for some groups. As long as the combined stochastic processes of birth and migration create some groups that have all species but no mutants, those groups will be favored by group-level selection. The combination of (rare) stochastic events with group-level selection can thus maintain cooperative interactions over evolutionary time scales. In nature, many mixed biofilms grow by a mechanism of “co-colonization” that resembles this process: one species often plays the role of the initial colonizer and other, mutualistic species later join [30]. That said, single-cell migration is not the only possible mechanism by which dispersal can occur in bacterial communities. This raises the question of how different migration modes may contribute to preventing mutational meltdown in multispecies communities under different fragmentation modes. For example, individuals may disperse in groups of relatives, as in the case of myxobacteria [75]. This mode of migration, termed “budding dispersal”, has a strong effect on the persistence of defectors [76–79]. Pichugin, Gokhale, Garcia, Traulsen, Rainey [80] explored the effect of four different modes of migration on the evolution of cooperation in a multilevel selection framework, and found that altruism is favored over defection when the mode of migration involves higher coordination between individuals. Although for the sake of simplicity we did not explore this question in our model, it would be interesting to consider how modes of migration interact with modes of fragmentation in preventing mutational meltdown in future work.

Strategies that intensify the effectiveness of group-level selection relative to individual-level selection can provide alternative ways to eliminate mutant cells. If larger groups are more likely than smaller ones to fission and produce offspring, group size becomes favored by multilevel selection. Since groups with fewer mutants grow faster, size-dependent fragmentation rates allow large, species-diverse groups to persist in the face of high mutation rates. Note, however, that in some circumstances larger group sizes may be less conducive to cooperative interactions [81–83]. Such group-size effects could be particularly relevant in cases where the effects of cooperation are nonlinear with the number of cooperators [84, 85], which is not the case in our model. Such nonlinear effects could make cooperation both more or less likely to evolve, depending on how they affect the relative balance between within- and between-group selection.

Migration and size-dependence are only two plausible solutions to the dilemma of fragmentation in multispecies communities. They are instructive in that each resolves the dilemma by relaxing one of the two conflicting selection pressures, but alternative solutions are, of course, entirely possible. One example is the segregation of cooperators and defectors during fission events, also known as associative splitting [52, 86–90]. In fact, when Kondrashov [6] first proposed that small propagule size reduces mutation load, he pointed out that the effect will be most effective when mechanisms are in place to ensure that the propagules are as homogeneous as possible. Hence, mutation load is minimized when only very related cells are recruited to form a propagule (i.e., associative splitting). Multicellular collectives are able to increase relatedness between propagule cells by creating segregated germ lines. Such complex mechanisms, however, are not required to achieve associative splitting. Even simple aggregations of cells can ensure that their propagules are maximally related by maintaining spatial structure. If daughter cells remain close to their parents, then group fragments that break off from the mother group will be highly homogeneous. This process could potentially allow large daughter groups to eliminate mutation load, however such groups would presumably also be homogeneous in terms of their species composition. Another simple mechanism is differential adhesion, where each species remains tightly attached to one (or very few) of the other species, which could produce multispecies propagules.

By incorporating migration and size-dependent fission rates, we observed that cooperative interactions can be maintained even for multi-species communities and for very high cooperation costs and mutation rates. Both migration and size-dependent fission are simple and realistic processes that are present in many natural microbial communities. Hence, our model suggests that cross-species cooperative interactions could potentially be stable in natural systems. This result stands in contrast to the view that cooperation is likely to be unimportant in microbial communities (e.g. [91]).

We also studied which fragmentation modes could be expected to evolve under multilevel selection. Experiments in a variety of species—including *Chlamydomonas reinhardtii* [92, 93], *Saccharomyces cerevisiae* [93], and *Pseudomonas fluorescens* [94]—show that the mode of fragmentation can rapidly evolve under selection pressure. Our simulation results suggest that strategies that maximize community productivity are evolutionary attractors.

The results we discussed are facilitated by the stochastic nature of our model. When newly born, small groups are created, sampling variation allows for the birth of groups that consist entirely of wild-type cells. In an infinite-size continuous limit, there would *always* be some fraction of mutants in every group. Because mutants grow faster than wild-type cells, even small fractions of mutants could pose challenges to the persistence of the community. Similarly, for the multi-species case, in the continuous limit there would *always* be some fraction of individuals of every species, which would render the penalty on small group production much smaller. To fully capture these effects it is thus essential to use an individual based modelling framework as we did here.

We can contrast our model to previous studies on fragmentation modes. For single-species groups, we recover the classic result of Kondrashov [6] (which has since been expanded by many other authors, e.g. [7, 19, 20]), viz. that by producing small propagules, single-species groups are able to persist in the presence of high mutation rates. Recently Pichugin, Peña, Rainey, Traulsen [5], Pichugin, Traulsen [28], and Pichugin, Park, Traulsen [29] developed an analytical framework which they used to calculate the optimal fragmentation mode for simple single-species groups in the absence of mutations. Their modelling approach is very different form ours, but is used to address similar questions. Despite the differences between the approaches, when we explicitly set out to compare our model to one of the findings from Pichugin, Peña, Rainey, Traulsen [5]—which shows that the optimal fragmentation mode depends on the rate functions used for cell birth and death—we found a similar result (Fig I in S5 Text). This indicates that the results from these approaches are not contradictory but complementary. The main strengths of Pichugin et al.’s approach are that it permits a full analytical treatment and that it incorporates all possible partitions. The main strength of our multilevel selection framework is that it is highly versatile: not only can we combine these different strands of the previous literature in a single modelling framework, but in addition we can use it to study multi-species groups, a topic which has received little attention so far. The versatility of our model also makes it easy to extend it in future work to further investigate how more complicated rate functions (e.g., nonlinear size-dependence in fission rate or different types of fission-associated costs) can explain the variety of fragmentation modes in nature.

## Supporting information

S1 Text List of Parameters. Contains a list of parameters and illustrates the functional forms of the cell- and group-level birth and death rates.
(PDF)

S2 Text Equilibrium group size and composition. Discusses how the number of groups, group size, and fraction of wild-type cells at equilibrium vary between different fragmentation modes.
(PDF)

S3 Text Alternative functional forms for rates. Explores the consequences of some alternative choices of functional form for group extinction rate and cell death rate.
(PDF)

S4 Text Effect of migration and of size-dependent fragmentation on maximum mutation rate. Illustrates the effect of size-dependent fragmentation rate and migration rate on the resilience of multispecies communities to mutational meltdown.
(PDF)

S5 Text Comparison with the results of Pichugin et al. Shows that our model is compatible with some results that were previously reported in the literature.
(PDF)
