# Supplementary material for: Multilevel selection favors fragmentation modes that maintain cooperative interactions in multispecies communities
Source: PLoS Comput Biol. 2021 Sep 13;17(9):e1008896. doi: 10.1371/journal.pcbi.1008896 (PMC8460008; doi:10.1371/journal.pcbi.1008896)
Supplement: S1 Text — Contains a list of parameters and illustrates the functional forms of the cell- and group-level birth and death rates. (PDF) [file pcbi.1008896.s001.pdf]

S1 List of parameters

Table A lists the parameters used in the model and their interpretation, and indicates the default values used for producing all figures (unless otherwise indicated in the text). Fig A (next page) shows the functional forms of all rate functions used in the main text.

Table A: List of parameters and default values that were using for producing figures.

Parameter		Default value
Parameters affecting community complexity		
m	Initial (maximum) number of species	1
Parameters affecting mutation and migration		
μ	Rate of mutation	0.001
ν	Rate of migration	0
Parameters affecting cell-level rates		
γ	Cost of cooperation	0.01
K_{cells}	Within-group carrying capacity	100
Parameters affecting group-level rates		
B_0	Minimum fission rate	0.05
σ	Slope of group size-dependence for fission rate	0
K_{total}	Scales the total number of cells in the community	10^5
Parameters affecting mode of fragmentation		
n	Fractional offspring number	(0, 1]
s	Fractional offspring size	(0, 0.5]

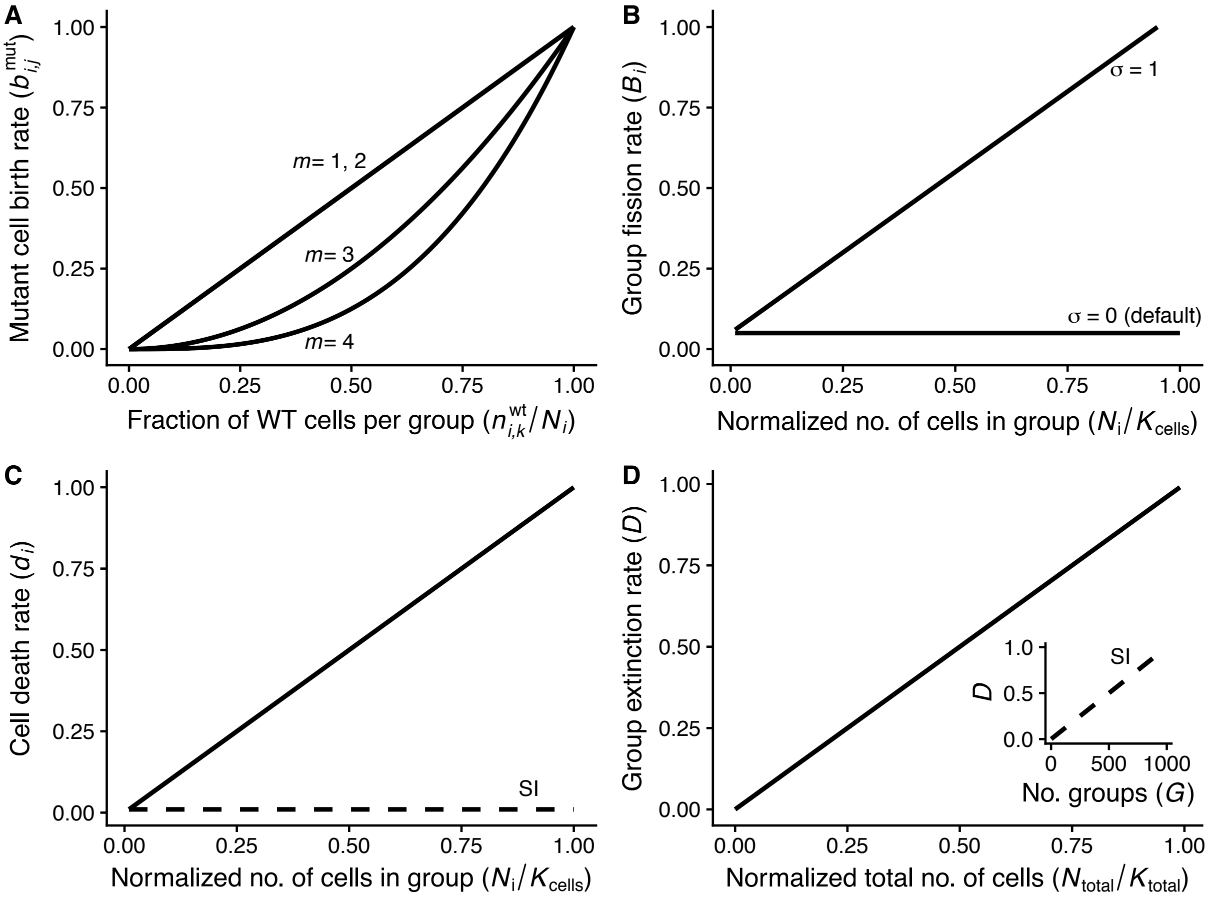

Fig A: Functional forms for cell- and group-level birth rates (top row) and death rates (bottom row). **Panel A:** cell birth rate (Eq 1, main text); **Panel B:** group fission rate (Eq 3, main text); **Panel C:** cell death rate (solid line from Eq 2, main text; dashed line from Eq S3.3 in S3 Text); **Panel D:** group extinction rate (solid line from Eq 2, main text; dashed line in inset from Eq S3.1 in S3 Text). Dashed lines indicate some of the alternative functional forms explored in this Supplemental Information. Parameters: $K_{groups} = 10^3$; all other parameters are set to the default values (table A), unless otherwise specified. For panel **A**, all groups, regardless of species, were assumed to have the same fraction of wild-type cells.
