# Supplementary material for: Multilevel selection favors fragmentation modes that maintain cooperative interactions in multispecies communities
Source: PLoS Comput Biol. 2021 Sep 13;17(9):e1008896. doi: 10.1371/journal.pcbi.1008896 (PMC8460008; doi:10.1371/journal.pcbi.1008896)
Supplement: S2 Text — Discusses how the number of groups, group size, and fraction of wild-type cells at equilibrium vary between different fragmentation modes. (PDF) [file pcbi.1008896.s002.pdf]

S2 Equilibrium group size and composition

At steady state, the composition of groups depends on the fragmentation mode. Fig B (*top*) shows the group-size distribution for each of the three fragmentation archetypes. With complete fragmentation, most groups are very small. With single-cell reproduction, some groups are large and some are small; this matches the intuition of large groups producing unicellular offspring. Finally, under binary fission, there is a comparatively uniform group size distribution, although higher mutation rates tend to lead to smaller group sizes when compared to lower mutation rates.

When the mutation rate is low, the frequency of wild-type cells tends to be very high across fragmentation modes; however, high mutation rates lead to a bimodal distribution where two large classes of groups exist: some groups have mostly wild-type cells and some groups have mostly mutant cells (Fig B, *middle*). The mutant-rich groups can even become more frequent than the wild-type-rich groups for some fragmentation modes (e.g., binary fission).

Across reproduction modes, there is a nonlinear relation between group size and fraction of wild-type cells. Very small groups often have high wild-type frequencies (which makes sense, because very small groups with high fractions of mutant cells tend to die off rapidly). If we disregard very small groups, there is a positive relation between group size and wild-type frequency (Fig B, *bottom*). This can be understood as follows: if new groups contain mutant cells at birth, these mutants will rapidly proliferate; as a result these groups will go extinct before they can reach a large size. Only groups that contain no mutants at birth can reach the carrying capacity (*de novo* mutations are rare, so these groups remain shielded from mutants from some time, allowing them to grow large before these mutants arise). As a result of these dynamics, mutant cells primarily occur in groups of intermediate size: these are groups that were recently born, where mutants have started to proliferate, but that have not yet gone extinct due to mutational meltdown.

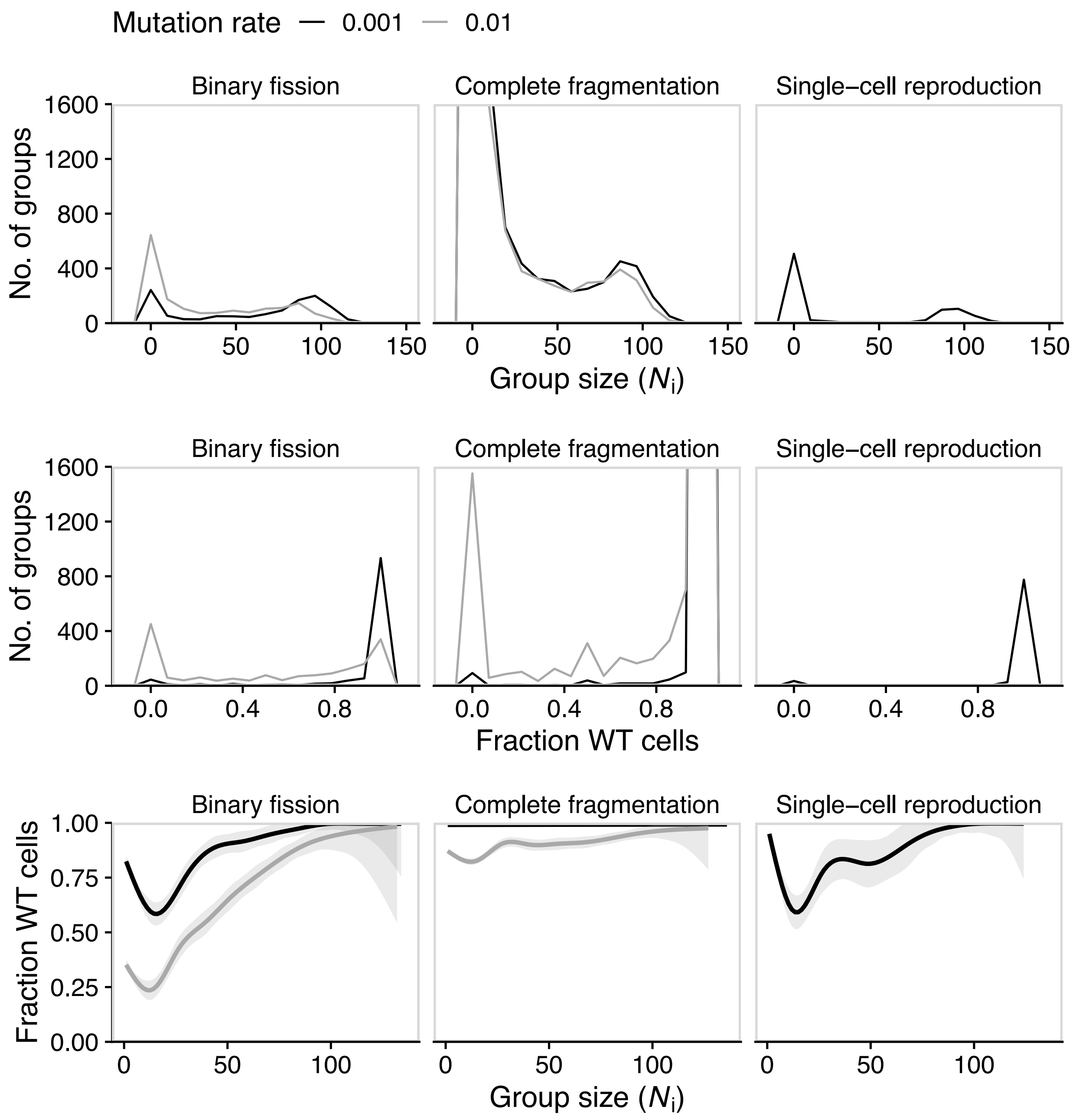

Fig B: Group size and composition at steady state. The figure shows the distribution of group sizes (*top*) and of the frequency of wild-type cells (cooperators) per group (*middle*), as well as the relation between these two properties (*bottom*), for the three different archetype fragmentation modes: binary fission ($s = 0.5, n = 0.5$, *left*), complete fragmentation ($s = 0.05, n = 0.95$, *middle*), and single-cell reproduction ($s = 0.05, n = 0.05$, *right*). Each line shows data from 10 replicate pooled together, for $\mu = 0.001$ (black) or $\mu = 0.01$ (grey) and $m = 2$. For the top and middle rows, the lines are frequency polygons (and the y-axis was cut at 1600 for clarity); for the bottom row, the lines are GAM smoothers accompanied by 95% confidence intervals.
