# Supplementary material for: Multilevel selection favors fragmentation modes that maintain cooperative interactions in multispecies communities
Source: PLoS Comput Biol. 2021 Sep 13;17(9):e1008896. doi: 10.1371/journal.pcbi.1008896 (PMC8460008; doi:10.1371/journal.pcbi.1008896)
Supplement: S3 Text — Explores the consequences of some alternative choices of functional form for group extinction rate and cell death rate. (PDF) [file pcbi.1008896.s003.pdf]

S3 Alternative functional forms for rates

The birth/death and fission/extinction rate functions used in the main text were chosen based on their simplicity, subject to the constraints that they should be biologically plausible and computationally feasible (Fig A in S1 Text). The choices in the main text are not necessarily the only reasonable options. In this section, we explore the consequences of some alternative choices: (1) a group extinction rate that increases with the number of groups rather than the total number of cells in the community; (2) a group extinction rate that decreases with group size, in addition to increasing with the total number of cells in the community; (3) a constant cell death rate.

Extinction rate increases with number of groups As the community grows, the extinction rate (Eq 4 in the main text) increases, which maintains the number of cells bounded at some upper limit. In the main text, we focused on the case in which the extinction rate increases with the number of *cells* in the community. Alternatively, it is conceivable that rate could instead increase with the number of *groups* in the community. This could be the case, for example, if the number of groups were bounded by the number of patches in which groups can reside. The simplest functional form that incorporates this kind of density-dependence is the following linear equation:

$$D = \frac{G}{K_{\text{groups}}}, \tag{S3.1}$$

where the parameter K_{groups} roughly scales the total number of groups in the community (see inset in Fig A, panel D, S1 Text). This kind of density-dependence provides qualitatively similar results to the one explored in the main text. For example, it is still the case that increasing the number of species moves the productivity optimum away from complete fragmentation and toward binary fission, reflecting a trade-off between resistance to mutational meltdown at low offspring sizes and the maintenance of mutualistic interactions at high offspring sizes (Fig C).

Extinction rate decreases with group size We also explored the possibility that the extinction rate, besides increasing with the number of cells in the community, also decreases with the number of cells in the group, such that smaller groups have higher extinction rates. Note that this small-group penalty is in addition to the effect that groups with small numbers of cells have higher risks of stochastic extinction. Biologically, it could represent, for example, increased risks of predation for small groups. We assume the

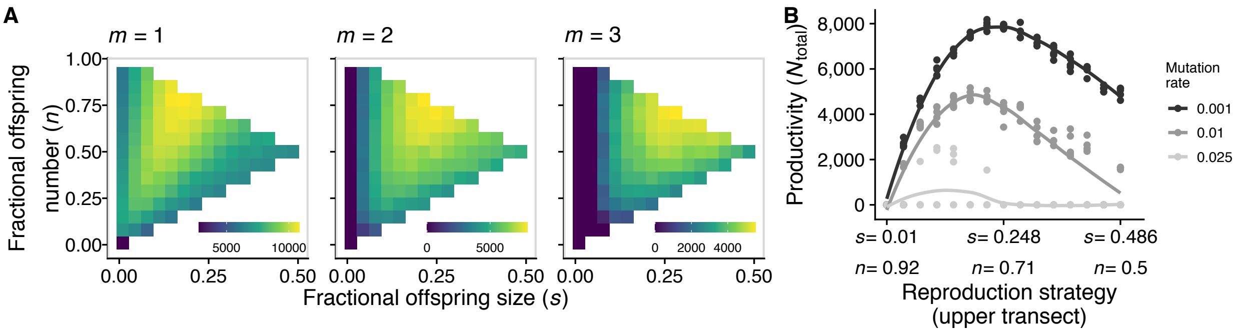

Fig C: When extinction rate grows with number of groups instead of number of cells, the main qualitative model result does not change: increased community complexity shifts the productivity peak away from small bottlenecks and toward binary fission. **Panel A:** The color indicates equilibrium community productivity (N_{total}). As the number of species (m) increases, the strategy that maximizes N_{total} moves rightward along the upper transect of the strategy space. **Panel B:** Some strategies that do well with small μ (large offspring) are not viable when μ is large (shown here with $m = 2$). Solid lines are LOESS smoothers. Parameters: $K_{\text{groups}} = 10^3$; all other parameters are set to the default values (table A).

following relationship:

$$D_i = \frac{N_{\text{total}}}{K_{\text{total}} N_i}, \tag{S3.2}$$

representing an extinction rate that increases linearly with group size (for a constant value of N_{total}) and increases linearly with N_{total} (for a constant value of N_i). In this case, the results are again generally similar to the case in the main text; one difference is that, when complexity increases, the shift in the productivity peak away from small bottlenecks and toward binary fission is less pronounced (Fig D).

Death rate does not depend on group size In the standard cell death rate scenario (Eq 2 in the main text, solid line in Fig A, panel C in S1 text) the cell death rate depends on the group size (i.e., density dependence) and thus on the fragmentation mode: following a fission event, cells have low death rates in the complete fragmentation case and high death rates in the binary fission case. (For the single-cell reproduction case, half of the cells will have high death rates and the other half will have low death rate.) Hence, the shape of the cell death rate functional form seems important in driving some of our results. To explore this possibility further, we investigated the consequences of using a constant, density-independent cell death rate (dashed line in Fig A, panel C in S1 text):

$$d = \frac{1}{K_{\text{cells}}}. \tag{S3.3}$$

Without any density-dependence on the death rate function, groups could potentially grow without

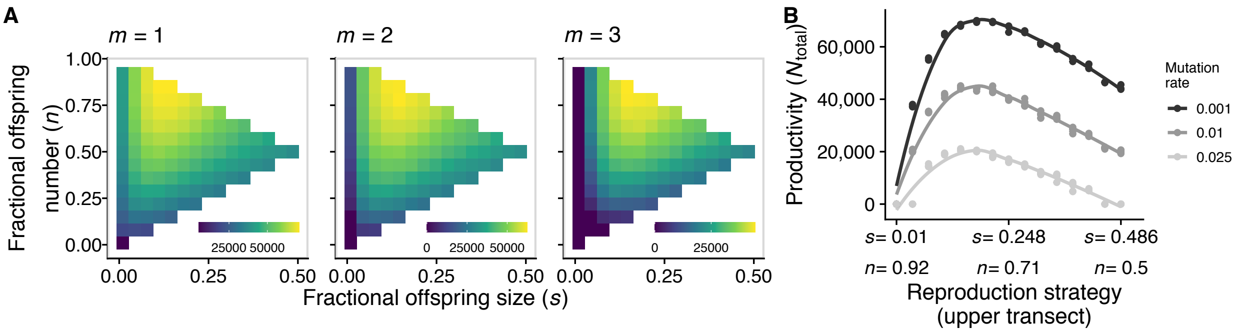

Fig D: When extinction rate decreases with group size in addition to increasing with the number of cells, the effect of increased community complexity (i.e., the shift of the productivity peak away from small bottlenecks and toward binary fission) is less pronounced. **Panel A:** The color indicates equilibrium community productivity (N_{total}). As the number of species (m) increases, the strategy that maximizes N_{total} moves slightly rightward along the upper transect of the strategy space (shown here with $\mu = 0.01$). **Panel B:** Some strategies that do well with small μ (large offspring) are less viable when μ is large (shown here with $m = 3$). Solid lines are LOESS smoothers. Parameters: $K_{\text{total}} = 10^4$; all other parameters are set to the default values (table A).

bounds, which would be biologically unrealistic and computationally unfeasible. To address this problem, we force all groups to immediately undergo fission when they reach the critical size K_{cells}.

With a density-independent death rate, we observe that the total productivity is similar for all fragmentation modes, even though the total number of groups is highest for the complete fragmentation mode (Fig E). Because of this difference relative to the results in the main text (Fig 2, main text), we explored the implications of a constant death rate to a deeper extent than those of the previously-described alternative rate functions.

Overall, we find that our conclusions are, to a large extent, qualitatively similar to those in the main text. It is still the case that increasing the number of species moves the productivity peak rightward along the strategy space (Fig F, panel A, B). However, there is one important difference: without a density-dependent death rate, the effect of the fractional offspring number (n) on productivity is very small (Fig F, panel A), meaning that there is not much difference between the complete fragmentation archetype and the single-cell fragmentation archetype. This makes sense because cells no longer derive an advantage from being in smaller groups.

Moreover, we investigated the effect of reproduction strategy on resistance to mutational meltdown (similar to Fig 5 in the main text). The results are no different from those in the main text: it is still the case that the more species a community has, the more sensitive it is to mutational-meltdown (Fig F, panel E). Moreover, the optimal fragmentation mode moves rightward in strategy space as the number of species increases (Fig F, panel E). Both can again be explained by a trade-off between the need to purge mutants

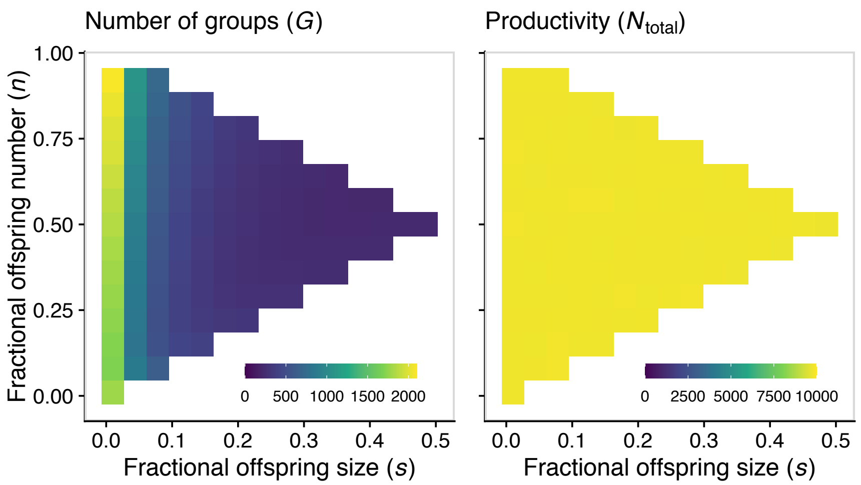

Fig E: Same as Fig 2 (main text), but in the case where there is no density dependence at the individual level (death rate given by Eq S3.3). In this case the total productivity is the same for all fragmentation-modes; the number of groups is maximized for the complete fragmentation mode. Parameters: $K_{\text{total}} = 10^4$, $\mu = 0$; all other parameters set to the default values (table A in S1 Text).

cells (requiring small offspring sizes) and the need to contain cells of all species (requiring larger offspring sizes).

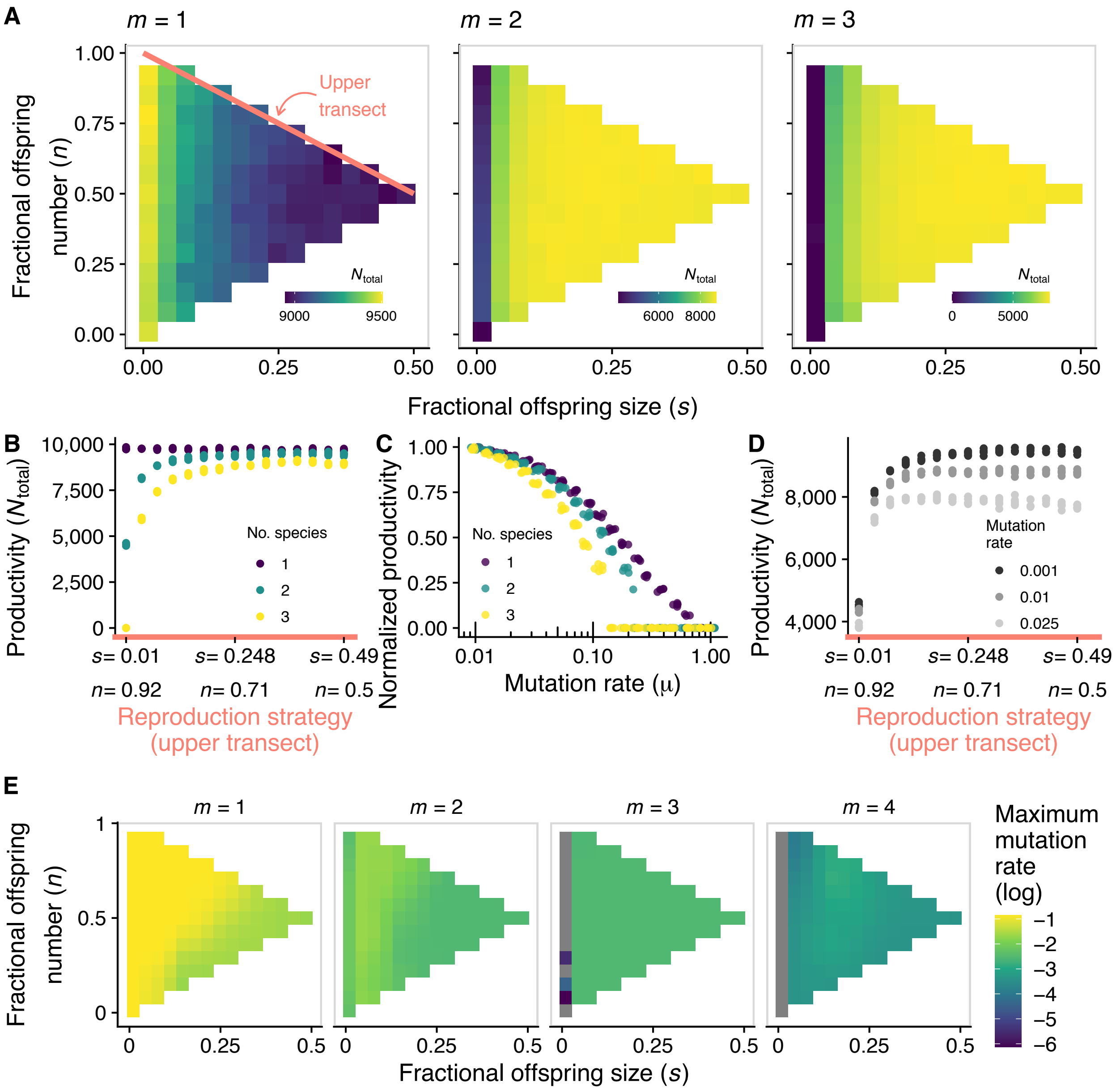

Fig F: When the cell death rate is independent of cell size, the effect of increased community complexity (i.e., the shift of the productivity peak away from small bottlenecks and toward binary fission) is still present. **Panel A:** The color indicates equilibrium community productivity (N_{total}). As the number of species (m) increases, the strategy that maximizes N_{total} moves rightward along the upper transect (pink line) of the strategy space. **Panel B:** Equilibrium productivity as a function of strategies along the upper transect of the strategy space (corresponding to the pink line from panel **A**). **Panel C:** Equilibrium productivity decreases with mutation rate (μ); this decrease is faster for higher numbers of species. **Panel D:** For large-offspring strategies (toward the right side of the x-axis), there is a decrease in productivity as the mutation rate increases. **Panel E:** Each subpanel shows, for each position in the strategy space, the maximum mutation rate a population can experience before going extinct (for details on implementation and interpretation, see the caption of Fig 5 in the main text). Parameters: $K_{\text{total}} = 10^4$; all other parameters are set to the default values (table A in S1 Text).
