# Supplementary material for: Multilevel selection favors fragmentation modes that maintain cooperative interactions in multispecies communities
Source: PLoS Comput Biol. 2021 Sep 13;17(9):e1008896. doi: 10.1371/journal.pcbi.1008896 (PMC8460008; doi:10.1371/journal.pcbi.1008896)
Supplement: S4 Text — Illustrates the effect of size-dependent fragmentation rate and migration rate on the resilience of multispecies communities to mutational meltdown. (PDF) [file pcbi.1008896.s004.pdf]

S4 Effect of migration and of size-dependent fragmentation on maximum mutation rate

In section 3.2.1 (main text), we showed that size-dependent fragmentation rate and migration can allow multispecies communities to prevent mutational meltdown (Fig 5, main text). The same results are shown below, in Fig G for a broader range of values of σ (slope of size-dependence), and in Fig H for a broader range of values of ν (migration rate).

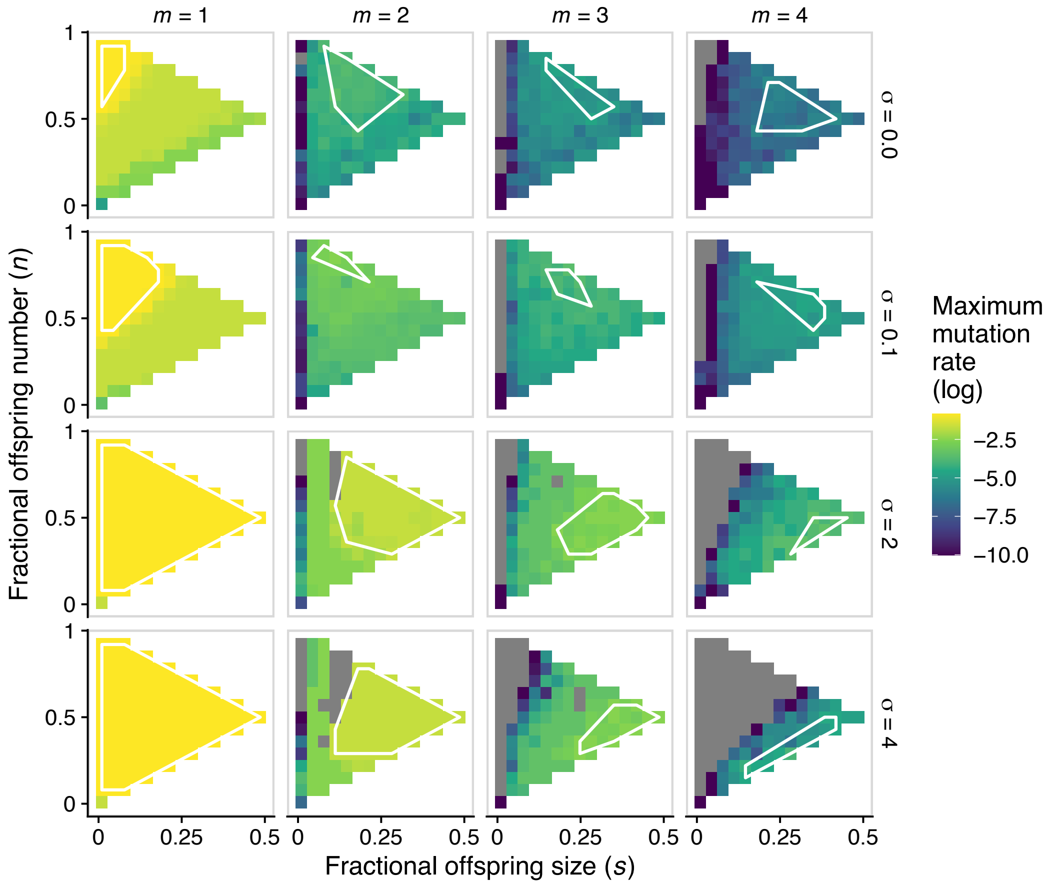

Fig G: Size-dependent fragmentation rate allows multispecies communities to resist mutational meltdown. Each panel shows, for each position in the strategy space, the maximum mutation a population can experience before going extinct (similar to Fig 3E and Fig 5, in the main text). Columns (from left to right) depict increasing number of species. Rows (from top to bottom) depict increasing slope of size-dependence. Grey squares correspond to communities for which the maximum mutation rate is outside the range of our simulations or numerical errors. The white polygons are convex hulls containing the five highest values of maximum mutation rate (to help guide the eye). For each value of mutation rate, we assessed ten replicates per fragmentation mode; the figure shows the mean (across replicates) of the logarithm of maximum mutation rate. Parameters: all parameters are set to the default values, unless otherwise indicated (table A).

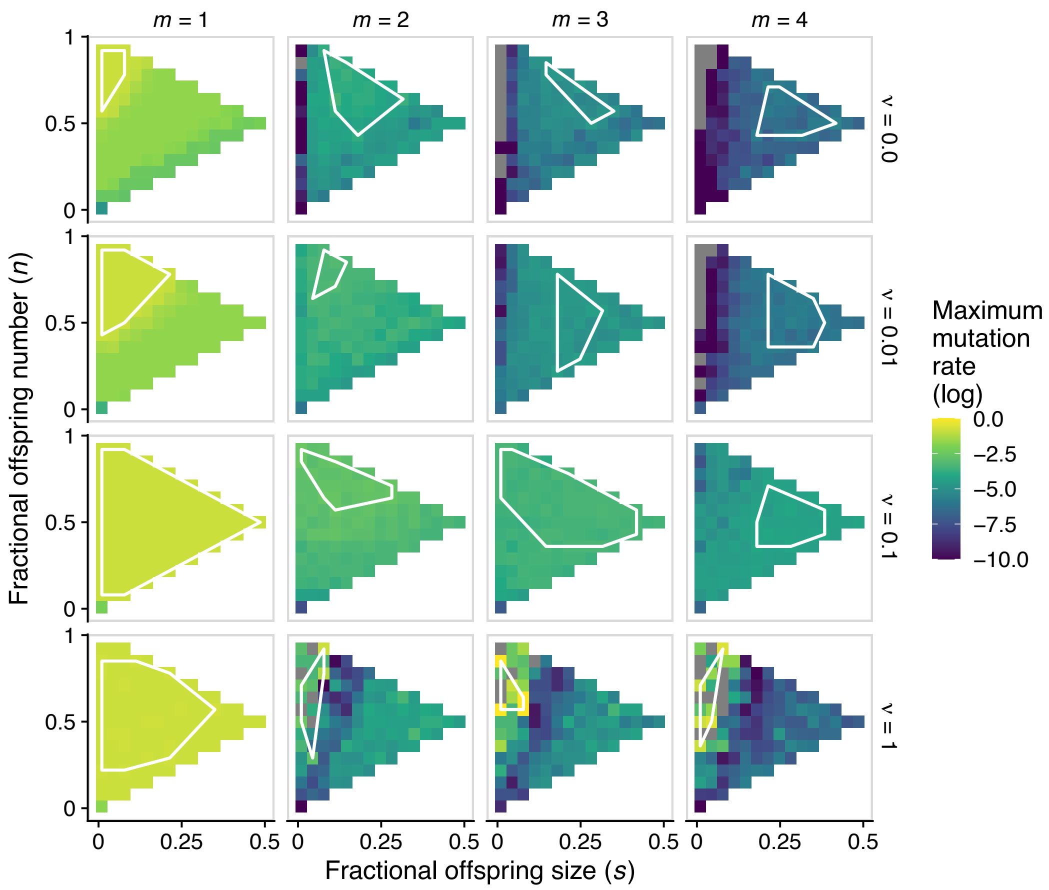

Fig H: Low to intermediate migration rates allow multispecies communities to resist mutational meltdown. Each panel shows, for each position in the strategy space, the maximum mutation a population can experience before going extinct (similar to Fig 3E and Fig 5, in the main text). Columns (from left to right) depict increasing number of species. Rows (from top to bottom) depict increasing migration rates. Grey squares correspond to communities for which the maximum mutation rate is outside the range of our simulations or numerical errors. The white polygons are convex hulls containing the five highest values of maximum mutation rate (to help guide the eye). For each value of mutation rate, we assessed ten replicates per fragmentation mode; the figure shows the mean (across replicates) of the logarithm of maximum mutation rate. Parameters: all parameters are set to the default values, unless otherwise indicated (table A).
