# Supplementary material for: Multilevel selection favors fragmentation modes that maintain cooperative interactions in multispecies communities
Source: PLoS Comput Biol. 2021 Sep 13;17(9):e1008896. doi: 10.1371/journal.pcbi.1008896 (PMC8460008; doi:10.1371/journal.pcbi.1008896)
Supplement: S5 Text — Shows that our model is compatible with some results that were previously reported in the literature. (PDF) [file pcbi.1008896.s005.pdf]

S5 Comparison with the results of Pichugin et al.

In Pichugin, Peña, Rainey, and Traulsen [1], cells within group i grow with a birth rate $b(N_i) = 1 + Mg(N_i)$, where $g(N_i) = [(N_i - 1)/(N_{\max} - 2)]^{\kappa}$. Here, $N_{\max}$ is the maximum cell number in a group, M is the maximum benefit of group life, and κ is a "complementarity" parameter that measures how each additional cell increases the benefit. When the number of cells reaches $N_{\max}$, the group fissions according to a given fissioning strategy, or "partition". Pichugin and collaborators [1] tested all mathematically possible partitions for a given $N_{\max}$, and measured their group-level fitness. Among other results, they found that the complementarity parameter κ is one of the main determinants of group-level fitness. Overall, if there are diminishing returns to the benefit of additional cells ($\kappa \ll 1$), binary fragmentation is the most fit partition, but if there are increasing returns ($\kappa \gg 1$), unicellular propagule production is the most fit. Other parameters, such as maximum benefit M, have a smaller influence on group-level fitness.

Our framework is compatible with this result from [1]. We set the group extinction rate to zero, and the birth rate of a cell in group i to $b_i(N_i) = 1 + g(N_i)$, with $g(N_i) = [(N_i - 1)/(K_{\text{ind}} - 2)]^{\kappa}$. Finally, we set the group fission rate to zero if $N_i < K_{\text{ind}}$, else $B_i = B_0 = 10^6$. When we measure group growth rate we observe the same result as in [1] (Fig I, panel A). The same pattern is observed when measuring cell growth rate (Fig I, panel B), which makes sense because, as soon as group size has equilibrated, both cells and groups have to grow at the same rate.

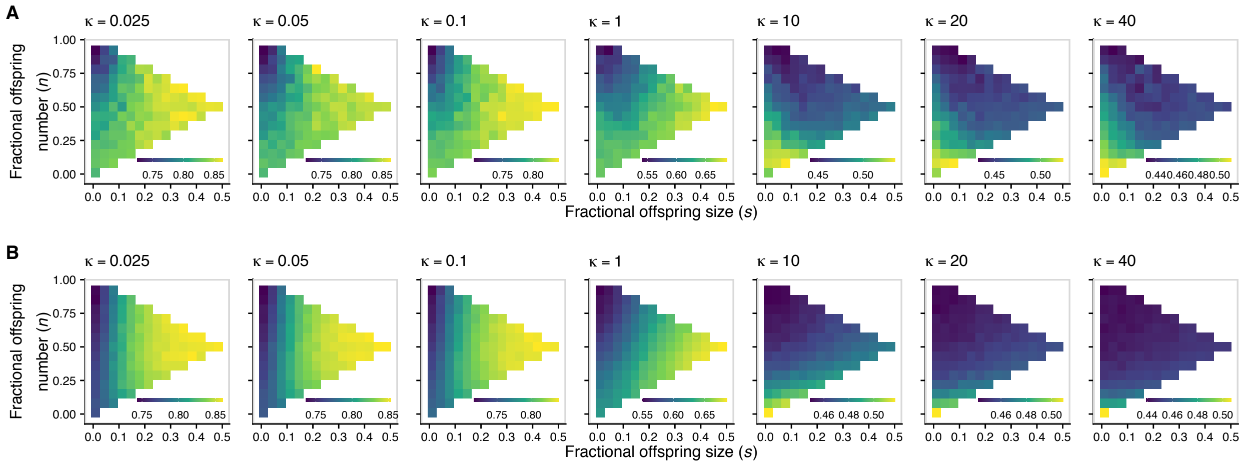

Fig I: In the absence of group-level density-dependence, if the cell birth rate increases with group size, the complementarity parameter κ determines which strategy maximizes fitness—measured either as group-level growth rate (**panel A**) or as cell-level growth rate (**panel B**). For $\kappa \ll 1$ (diminishing returns), binary fragmentation maximizes fitness, whereas for $\kappa \gg 1$ (increasing returns), single-cell reproduction maximizes fitness. The color scale indicates growth rate.

References

1. Pichugin Y, Peña J, Rainey PB, Traulsen A. Fragmentation modes and the evolution of life cycles. PLoS Computational Biology. 2017;13(11):e1005860.
